# Can ChatGPT help patients understand radiopharmaceutical extravasations?

**DOI:** 10.3389/fnume.2024.1469487

**Published:** 2024-11-06

**Authors:** Madeleine Alvarez

**Affiliations:** Independent Researcher, Cary, NC, United States

**Keywords:** radiopharmaceutical extravasation, SNMMI, diagnostic imaging, medical event reporting, AI in healthcare, nuclear medicine procedures, patient education AI

## Abstract

A previously published paper in the official journal of the Society of Nuclear Medicine and Molecular Imaging (SNMMI) concluded that the artificial intelligence chatbot ChatGPT may offer an adequate substitute for nuclear medicine staff informational counseling to patients in an investigated setting of ^18^F-FDG PET/CT. To ensure consistency with the previous paper, the author and a team of experts followed a similar methodology and evaluated whether ChatGPT could adequately offer a substitute for nuclear medicine staff informational counseling to patients regarding radiopharmaceutical extravasations. We asked ChatGPT fifteen questions regarding radiopharmaceutical extravasations. Each question or prompt was queried three times. Using the same evaluation criteria as the previously published paper, the ChatGPT responses were evaluated by two nuclear medicine trained physicians and one nuclear medicine physicist for appropriateness and helpfulness. These evaluators found ChatGPT responses to be either highly appropriate or quite appropriate in 100% of questions and very helpful or quite helpful in 93% of questions. The interobserver agreement among the evaluators, assessed using the Intraclass Correlation Coefficient (ICC), was found to be 0.72, indicating good overall agreement. The evaluators also rated the inconsistency across the three ChatGPT responses for each question and found irrelevant or minor inconsistencies in 87% of questions and some differences relevant to main content in the other 13% of the questions. One physician evaluated the quality of the references listed by ChatGPT as the source material it used in generating its responses. The reference check revealed no AI hallucinations. The evaluator concluded that ChatGPT used fully validated references (appropriate, identifiable, and accessible) to generate responses for eleven of the fifteen questions and used generally available medical and ethical guidelines to generate responses for four questions. Based on these results we concluded that ChatGPT may be a reliable resource for patients interested in radiopharmaceutical extravasations. However, these validated and verified ChatGPT responses differed significantly from official positions and public comments regarding radiopharmaceutical extravasations made by the SNMMI and nuclear medicine staff. Since patients are increasingly relying on the internet for information about their medical procedures, the differences need to be addressed.

## 1 Introduction

ChatGPT is an advanced language model that uses deep learning techniques and neural networks to analyze and produce human-like responses to posed questions (1). Recently, there has been substantial discussion surrounding the use of ChatGPT in medicine. With the rapid expansion of healthcare and increasing physician burnout exacerbated by the recent COVID pandemic, there has been much discussion of using AI assistants such as ChatGPT to aid in answering patient questions (2). While the utilization of ChatGPT in healthcare has been limited, it is possible that ChatGPT could enhance the overall efficiency and accuracy of the healthcare sector, particularly as AI science becomes increasingly more sophisticated.

With regards to ChatGPT's application to the field of nuclear medicine, Rogasch et al. provided ChatGPT with common patient inquiries regarding PET/CT studies and their associated reports (3). Experts then evaluated the AI responses to assess whether ChatGPT adequately addressed the inquiries and explained the reports. The authors concluded that ChatGPT may offer an adequate substitute for patient information given by nuclear medicine staff in the setting of PET/CT for Hodgkin lymphoma or lung cancer.

Radiopharmaceutical extravasations have been receiving increased interest in peer-reviewed articles and regulatory discussions for the past five years. Radiopharmaceutical extravasations refer to the inadvertent injection of a radiopharmaceutical into the surrounding tissue at the injection site, instead of the appropriate blood vessel (4). The U.S. Nuclear Regulatory Commission (NRC) has a policy that exempts all radiopharmaceutical extravasations from medical event reporting, even if they meet or exceed reporting criteria of other misadministrations (5). This exemption policy was reviewed as a result of a recent petition for rulemaking (6). In December 2022, the NRC accepted the petition, solicited public comments in writing, and commenced rulemaking to address the reporting of extravasations in April 2023 (7). Throughout this process, NRC has noted that the extravasation issue has generated an unusually high level of interest^1^^,^^2^.

Since the filing of the petition in 2020, patients and patient advocacy organizations have become increasingly interested in this topic. In February 2021, the Patients for Safer Nuclear Medicine (PSNM) coalition was formed to demand that patients get the information they need about extravasations, so their diseases are accurately diagnosed and treated. By February 2024, the coalition had thirty patient advocacy organization members representing thousands of patients and nine corporate partners^3^. A review of the PSNM website reveals over a dozen press releases, letters to the NRC^4^, and op-eds^5^^,^^6^.

In response to the petition and recently proposed rulemaking, over 600 public comments have been submitted to the NRC by patients, patient advocates, clinicians, physicists, medical societies, industry groups, private companies, and government agencies^7^^,^^8^.

Rogasch et al., determined that ChatGPT could offer an adequate substitute for informational counseling to patients in lieu of that provided by nuclear medicine staff regarding certain PET/CT studies and their reports. We used their published methodology to examine whether ChatGPT could also adequately substitute for informational counseling regarding radiopharmaceutical extravasation.

## 2 Materials and methods

### 2.1 Generating questions about extravasations

We formulated fifteen patient-oriented questions concerning radiopharmaceutical extravasations (Supplementary File S1) based on a thorough review of over 600 public comments submitted to the NRC. These comments came from diverse stakeholders, including patients, advocacy groups, healthcare professionals, and medical organizations. Our question selection process involved identifying recurring themes and topics of particular concern to patients and stakeholders. We prioritized topics based on frequency of mention across comments, perceived importance to patient understanding and safety, and relevance to patient experience throughout nuclear medicine procedures.

Using these key themes, we crafted questions covering a range of topics from basic definitions to potential health impacts and patient rights. We intentionally used simple, non-technical language to ensure accessibility to a general patient audience. The initial list was then reviewed and refined by nuclear medicine experts to ensure comprehensive coverage while avoiding redundancy. Although this process involved some subjective judgment, we believe it resulted in a representative and comprehensive set of patient-oriented questions that effectively captured the most salient concerns regarding radiopharmaceutical extravasations.

### 2.2 ChatGPT responses

We accessed OpenAI ChatGPT Plus in the form of GPT-4 in January 2024. Using the same methodology employed by Rogasch et al., all questions were entered as single prompts in separate chats. Each prompt was repeated twice using the regenerate-response function, resulting in 3 trials per prompt. In addition, ChatGPT was asked to provide references for its responses.

### 2.3 Rating process

Two nuclear medicine-trained physicians and one nuclear medicine physicist independently rated the ChatGPT responses using the rating scale previously published by Rogasch et al. (Table 1). One physician is a nuclear medicine-trained board-certified radiologist with over 30 years of clinical and research experience and over 12 publications on radiopharmaceutical extravasations. The other physician is an American Board of Radiology-certified radiologist with more than 40 years of experience in academic clinical and investigative radiology, including subspecialty certification in nuclear radiology. The nuclear medicine physicist has served for decades on the Medical Internal Radiation Dose (MIRD) Committee of the Society of Nuclear Medicine and Molecular Imaging (SNMMI) and has been a member of the NRC's Advisory Committee on the Medical Uses of Isotopes. Each evaluator rated the appropriateness and helpfulness of the responses and assessed the three responses for each question for inconsistency. Criteria ratings were designed to capture AI hallucinations since these can misinform and potentially cause harm. To ensure that neutral evaluations could not be generated, we assessed appropriateness and helpfulness with a 4-point scale. One physician checked and rated the validity of all references provided by ChatGPT.

**Table 1:** Criteria and categories used for rating.

Criterion	Description
Appropriateness	
1. Highly appropriate	Meeting standards of information given by medical staff in nuclear medicine department
2. Quite appropriate	Minor aspects incorrect or inconsistent
3. Quite inappropriate	Relevant aspects inconsistent
4. Highly inappropriate	Major aspects incorrect; potentially harmful
Helpfulness	
1. Very helpful	Comprehensive and likely to fully answer patient's question
2. Quite helpful	Specific but lacking potentially helpful information
3. Quite unhelpful	Specific but lacking crucial information related to patient's question
4. Clearly unhelpful	Unspecific and lacking crucial information
Inconsistent between trials	
1. Irrelevant	Differences only in wording, style, or layout
2. Minor	Differences in content of response but none relevant to main content required to answer patient's question
3. Major	Some differences relevant to main content
4. Incompatible	Responses incompatible with each other
Validity of references	
1. Fully valid	Appropriate, identifiable, and accessible source
2. Appropriate but outdated	Appropriate reference but outdated uniform resource locator or only generic references
3. Appropriate, incorrectly cited, but possible to find	Appropriate reference with incorrect bibliographic data but still possible to find
4. Invalid	Invalid reference that cannot be found (hallucinations)
N/A	Responses were generated using generally available medical information, and no references were provided.

Interobserver agreement among the three evaluators was assessed using the Intraclass Correlation Coefficient (ICC). The ICC was calculated across all evaluation criteria (appropriateness, helpfulness, and inconsistency) to determine the overall reliability of ratings among the evaluators.

### 2.4 Statistical analysis

For each question posed, three ChatGPT responses were generated and evaluated by three evaluators using three criteria: appropriateness, helpfulness, and inconsistency between trials.

Appropriateness and helpfulness were assessed using an ordinal, categorical scale ranging from 1 (appropriate or helpful response) to 4 (not appropriate or unhelpful). Employing the same statistical methods as in previously published paper by Rogasch et al. (3)., the majority rating among the three evaluators was calculated for each response to determine the overall appropriateness and helpfulness scores for each question. When three different ratings were given, the middle category was chosen. The median of these majority ratings was then used to represent the overall appropriateness and helpfulness scores for that question.

Inconsistency between trials was evaluated by having each evaluator assess the three responses generated for each question. The majority rating among the three evaluators was used to determine the inconsistency score for each question. Following the same approach as with appropriateness and helpfulness, when the evaluators gave different ratings, the middle category was chosen.

Validity of references was assessed by a single physician who checked and rated all references provided by ChatGPT.

Interobserver agreement among the three evaluators was assessed using the ICC (Supplementary File S2). The ICC was calculated using a two-way random-effects model with absolute agreement, denoted as ICC(2,1). The following variables were used in the calculation: Mean Square for Rows (MSR), Mean Square for Columns (MSC), Mean Square for Error (MSE), the number of raters (k), and the number of subjects (n). The formula for ICC(2,1) is:

ICC(2,1) = (MSR—MSE)/[MSR + (k - 1) * MSE + (k/n) * (MSC—MSE)].

In summary, three overall median ratings were calculated, one for each of the evaluation criteria: appropriateness, helpfulness, and inconsistency. These overall median ratings provide a measure of ChatGPT's performance for each criterion across all fifteen questions. The ICC was used to assess the overall interobserver agreement among the evaluators.

### 2.5 Quantitative analysis of response structure

To provide analysis of structural characteristics of ChatGPT-generated text, we conducted a quantitative assessment of the responses. We calculated the average response length, identified the shortest and longest responses, determined the average number paragraphs per response, assessed the percentage of responses using bullet points or numbered lists, and computed the average number of list items when used. This quantitative analysis offers insights into ChatGPT's text generation patterns.

## 3 Results

The fifteen questions and a summary of the ratings are in Table 2. Ratings by individual evaluator for each of the forty-five responses, ratings for the inconsistency across responses, and a rating regarding ChatGPT-identified references can be found in Supplementary File S3.

**Table 2:** Evaluation of radiopharmaceutical extravasation responses based on appropriateness, helpfulness, consistency, and reference validity.

Questions (general)	Median score (*n* = 3)			
	Appropriateness	Helpfulness	Inconsistency between responses	Validity of references
1. What are radiopharmaceutical extravasations?	1	1	2	1
2. How frequently do extravasations occur during radiopharmaceutical administrations?	2	3	3	1
3. How should clinicians address a suspected radiopharmaceutical extravasation?	1	1	2	1
4. How important is it to mitigate a radiopharmaceutical extravasation quickly?	1	1	2	1
5. Who should receive a report of a radiopharmaceutical extravasation?	1	1	2	N/A
6. Can radiopharmaceutical extravasations be prevented or minimized?	1	1	2	1
7. Should a patient be concerned about diagnostic radiopharmaceutical extravasation?	1	1	2	1
8. How does a radiopharmaceutical extravasation affect a patient?	1	1	1	1
9. Are radiopharmaceutical extravasations harmful?	2	2	2	1
10. Are there any long-term effects of radiopharmaceutical extravasation?	1	1	2	1
11. Are there any systemic effects of radiopharmaceutical extravasations?	1	2	2	1
12. Should a patient be told when they have been extravasated with a radiopharmaceutical?	1	1	1	N/A
13. Can a radiopharmaceutical extravasation affect a patient's diagnostic image?	2	1	3	1
14. How does an extravasation affect a patient's therapeutical radiopharmaceutical procedure?	1	1	2	N/A
15. Could a radiopharmaceutical extravasation alter the qualitative and quantitative results of a nuclear medicine procedure in a way that causes the patient to receive inappropriate or less than optimal care?	2	2	2	N/A
**Overall Median Rating**	**1**	**1**	**2**	**1**

### 3.1 Rating of appropriateness and helpfulness

The appropriateness rating of the responses for all fifteen questions was either 1 or 2, representing highly appropriate or quite appropriate. The overall appropriateness rating for the fifteen questions was 1.

The helpfulness rating of the responses for fourteen of the questions was either 1 or 2, representing very helpful or quite helpful. The helpfulness rating for 1 question was 3. The overall helpfulness rating for the fifteen questions was 1.

### 3.2 Rating of inconsistency

The inconsistency rating for eleven of the questions was 2, representing minor inconsistencies. The inconsistency rating for 2 questions was 1, representing irrelevant inconsistencies. For the remaining 2 questions, the inconsistency rating was 3, representing major inconsistencies. The overall inconsistency rating of the fifteen questions was 2.

### 3.3 Validity of references

ChatGPT used fully validated (appropriate, identifiable, and accessible) references in eleven of the fifteen questions. For the remaining four questions ChatGPT used generally available medical and ethical guidelines to generate its responses. No AI hallucinations were found.

### 3.4 Interobserver agreement

The ICC(2,1) for the two-way random-effects model with absolute agreement was found to be 0.72. According to the guidelines proposed by Cicchetti (1994)^9^^,^^10^, an ICC value between 0.60 and 0.74 indicates “good” reliability. Therefore, the obtained ICC value of 0.72 suggests that there was good overall agreement among the evaluators when rating the appropriateness, helpfulness, and inconsistency of the ChatGPT responses across all fifteen questions.

### 3.5 Structural analysis of responses

Our quantitative analysis of ChatGPT responses revealed consistent patterns in text structure. The average response length was 278 words (excluding references), with responses ranging from 121 to 434 words. Responses typically contained an average of 3 paragraphs, with bullet points and numbered lists treated as single paragraphs due to their cohesive nature. Notably, 91% of responses incorporated bullet points or numbered lists, averaging 5 list items when used. These findings indicate that ChatGPT generates moderately lengthy responses with a clear, organized structure, predominantly utilizing bullet points or numbered lists to present information.

## 4 Discussion

Rogasch et. al, found that ChatGPT could offer an adequate substitute for informational counseling to patients in lieu of that provided by nuclear medicine staff regarding certain PET/CT studies and their reports. In this work, we examined whether ChatGPT could offer an adequate substitute for informational counseling regarding radiopharmaceutical extravasations.

Three expert evaluators, members of the American College of Radiology, Society of Nuclear Medicine and Molecular Imaging, and Health Physics Society, rated the ChatGPT responses as appropriate and helpful with only minor inconsistencies across multiple responses to the same questions. The ICC rating of 0.72 suggests good overall agreement among the evaluators in their ratings. Furthermore, a review of the references used by ChatGPT to generate responses found no hallucinations. Eleven of the fifteen responses used fully validated references and four used generally available medical and ethical guidelines to generate responses.

AI is playing an increasing role in medically related searches with an AI overview being displayed frequently at the top of the search results page^11^^,^^12^ (Supplementary File S4). According to a recent survey of 2,000 U.S. adults^13^, more Americans trust social media and healthcare websites for advice over a medical professional, 94% trust AI to handle certain health-related tasks, and over half (52%) have consulted large language models like ChatGPT for medical diagnoses, reflecting the growing role of AI in personal healthcare decisions. With the increasing patient interest in radiopharmaceutical extravasations, several AI concerns need to be addressed.

AI systems, including ChatGPT, operate from data provided to the system, and therefore may be biased. Obermeyer et al. demonstrated that commercial prediction algorithms exhibited significant racial bias, and worsened care for patients (8). Bias in the training data can cause AI systems to produce biased responses. While an examination of bias in ChatGPT's algorithms was not included in the methodology employed by Rogasch et al., evaluators in our study made no comment of bias during their review of responses.

While the free version, GPT-3.5, may have been more readily available to patients in January 2024, we chose to follow the methodology from the previously published study by Rogasch et al., and used ChatGPT Plus in the form of GPT-4. Unlike GPT-3.5, GPT-4 provides references for evaluation.

Rogasch et al. stressed the importance of ensuring the quality of responses that ChatGPT provides. AI can produce potentially dangerous responses. ChatGPT can produce false, incoherent, or unrelated responses that are known as hallucinations^14^ (9). No responses were found to be potentially harmful or lacking crucial information.

Our evaluators generally found ChatGPT responses to be appropriate, helpful, and with minimal inconsistency. Two hundred eighty-three of the possible three hundred fifteen ratings were rated highly appropriate, quite appropriate, very helpful, quite helpful, or showed irrelevant or minor differences across responses. However, there were exceptions. Thirty-two responses were rated as quite inappropriate, quite unhelpful, or showed major differences across responses. Evaluators’ criticisms can be summarized in several themes.

Evaluators noted that certain ChatGPT responses were not clearly appropriate or helpful. For example, they cited extravasation rates in some responses that relied on data from areas outside of nuclear medicine. They noted that some ChatGPT responses recommended using cold compresses to mitigate the effects of radioactivity in tissue, while other responses recommended warm compresses. They noted that some ChatGPT responses suggested using vascular access tools other than those recommended by vascular access experts (10). And they also noted that ChatGPT used terminology that may be vague or confusing for a general patient (e.g., radiotoxic, critical structures).

Evaluators reported that some ChatGPT responses suggested that nuclear medicine practices routinely and effectively monitor for, identify, characterize, and mitigate radiopharmaceutical extravasations (10). The evaluators found no evidence to support these responses.

Evaluators also noted that, while ChatGPT accurately identified that extravasations can negatively affect the quality of diagnostic images, responses did not address their effect on quantification of images.

Beyond the criticisms identified by individual evaluators, we observed an overarching theme repeated throughout the ChatGPT responses. 41 out of 45 responses (91%) suggested that radiopharmaceutical extravasations can cause adverse effects, including local tissue reaction and radiation-induced tissue damage (Supplementary File S1). Public comments to the NRC broadly supported this theme. The American Society for Radiation Oncology, wrote that “[they] recognize that serious patient harm can occur if an injection of a radiopharmaceutical goes awry^15^.” Similarly, the University of Pennsylvania Office of Environmental Health and Radiation Safety detailed specific potential impacts, stating that extravasation could “potentially cause blistering, tissue damage/sloughing with or without necrosis and potential structural damage”, and that “potential harm…depends on the dose to the surrounding tissue^16^”. The National Institutes of Health commented that they would be in favor of the “prompt reporting of radiation safety-significant extravasations from high energy radiopharmaceuticals^17^.” Though these organizations advocate different approaches to medical event reporting, they all recognize that extravasation could be a patient safety issue.

Despite the well-known challenges associated with AI and medical uses previously discussed and addressed, we found ChatGPT's responses regarding radiopharmaceutical extravasations appropriate and helpful. However, some public positions and comments conflict with ChatGPT's responses and our experts’ evaluations.

For example, while the SNMMI acknowledged that extravasations can affect the quality and quantification of diagnostic imaging procedures^18^, they have also submitted other comments to the NRC. In these comments, SNMMI minimized the potential effects of radiopharmaceutical extravasation. When responding to NRC's request for what information should be provided to patients if extravasation is suspected, SNMMI stated, “Patients receiving diagnostic radiopharmaceuticals need not be concerned^19^.” SNMMI also stated that extravasations are infrequent and not severe. In regard to medical event reporting, SNMMI expressed concern that reporting will induce “radiation paranoia” and a “chilling effect” among patients^20^.

Because the SNMMI position, which likely represents the position of most nuclear medicine staff, conflicts with ChatGPT responses, we cannot claim ChatGPT currently offers an adequate substitute for informational counseling regarding radiopharmaceutical extravasations. Further research and interviews with the leaders of SNMMI and other organizations may be required to better determine why these differences exist and how they can be clarified or resolved to ensure patients receive accurate information.

Our study has two main limitations. First, we did not conduct a patient comprehension evaluation, which is crucial for assessing the effectiveness of AI-generated patient education materials. Second, our panel of evaluators did not include radiographers or nuclear medicine technologists, whose insights could provide valuable perspective on practical applicability. While this aligns with the methodology of Rogasch et al., future studies would benefit from including a broader range of healthcare professionals and directly assessing patient understanding. These limitations highlight important areas for future research in AI-assisted patient communication in nuclear medicine.

## 5 Conclusion

The ChatGPT responses received high ratings by experts and were judged to be a reliable resource for patient education. However, ChatGPT responses frequently differed significantly with official positions and public statements from some professional societies. For nuclear medicine practitioners and patients to benefit from AI, these differences must be resolved.

Once that happens, AI could potentially support patient-provider relationships by serving as a preliminary source of general information. This could allow patients to come to their appointments better informed about basic concepts, potentially leading to more productive discussions with their healthcare providers. Additionally, the availability of AI-based patient education could improve patient understanding of nuclear medicine procedures and allow them to better advocate for themselves and the quality of their care.

## Data Availability

The original contributions presented in the study are included in the article/Supplementary Material, further inquiries can be directed to the corresponding author.

## Footnotes

^1^https://www.nrc.gov/docs/ML2402/ML24025A055.pdf; page 48, lines 3-7.

^2^https://www.nrc.gov/docs/ML2410/ML24109A127.pdf; page 57, lines 20-23.

^3^ https://www.safernuclearmedicine.org/about-us.html

^4^ https://www.safernuclearmedicine.org/position-statements-recommendations.html

^5^ https://www.safernuclearmedicine.org/media.html

^6^ https://medtech-devices-startups.medicaltechoutlook.com/cxoinsight/-extravasation-101-what-is-it-how-can-you-protect-yourself-and-your-patients-nwid-3056.html0

^7^ https://medtech-devices-startups.medicaltechoutlook.com/cxoinsight/-extravasation-101-what-is-it-how-can-you-protect-yourself-and-your-patients-nwid-3056.html

^8^ https://www.regulations.gov/docket/NRC-2022-0218/comments

^9^ https://en.wikipedia.org/wiki/Intraclass_correlation

^10^ https://psycnet.apa.org/doiLanding?doi=10.1037%2F1040-3590.6.4.284

^11^Link to the search 'What are radiopharmaceutical extravasations?' in Google, displaying an AI overview.

^12^"Google Is Using A.I. to Answer Your Health Questions. Should You Trust It?" The New York Times, June 5, 2024.

^13^ https://www.usertesting.com/resources/reports/consumer-perceptions-ai-healthcare

^14^ https://academic.oup.com/eurheartj/article/45/5/321/7471676

^15^ https://www.regulations.gov/comment/NRC-2022-0218-0157

^16^ https://www.regulations.gov/comment/NRC-2022-0218-0163

^17^ https://www.regulations.gov/comment/NRC-2022-0218-0146

^18^ https://s3.amazonaws.com/rdcms-snmmi/files/production/public/SNMMI%20statement_final%20signed%20w%20letterhead%209-29-20.pdf

^19^ https://snmmi.org/common/Uploaded%20files/Web/Position%20Statements/SNMMI%20statement_final%20signed%20w%20letterhead%209-29-20.pdf

^20^ https://sites.snmmi.org/common/Uploaded%20files/Web/Advocacy%20and%20Initiatives/2023-09-07/NRC%20Comments%20on%20Extravasations%20Rulemaking%209-1-2023.pdf
