# Corrigendum: Can ChatGPT help patients understand radiopharmaceutical extravasations?

**DOI:** 10.3389/fnume.2025.1534645

**Published:** 2025-05-29

**Authors:** Madeleine Alvarez

**Affiliations:** Cary Academy, Cary, United States

**Keywords:** radiopharmaceutical extravasation, SNMMI, diagnostic imaging, medical event reporting, AI in healthcare, nuclear medicine procedures, patient education AI

A Corrigendum on Can ChatGPT help patients understand radiopharmaceutical extravasations? By Alvarez M (2024). Front. Nucl. Med. 4:1469487. doi: 10.3389/fnume.2024.1469487

In the published article, the URLs for References 5, 6, and 7 were missing. The references and the added links appear below:

5. Misadministration Reporting Requirements. 45 Fed. Reg. (May 14, 1980): 31701–31705. Available from: https://tile.loc.gov/storage-services/service/ll/fedreg/fr045/fr045095/fr045095.pdf

6. U.S. Nuclear Regulatory Commission. Federal Register—Reporting Nuclear Medicine Injection Extravasations as Medical Events. (2022). p. 80474. Available from: https://www.federalregister.gov/documents/2022/12/30/2022-28356/reporting-nuclear-medicine-injection-extravasations-as-medical-events

7. NRC. secy-22-0043: petition for rulemaking and rulemaking plan on reporting nuclear medicine injection extravasations as medical events. (2022). Available from: https://www.nrc.gov/materials/miau/med-use-toolkit/reporting-nuclear-medicine-injection-extravasations.html

In the published article, there were errors in the links for footnotes 18, 19, and 20 and these did not resolve. The corrected footnote URLs appear below:

Footnote 18

https://snmmi.org/common/Uploaded%20files/Web/Position%20Statements/SNMMI%20statement_final%20signed%20w%20letterhead%209-29-20.pdf

Footnote 19

https://sites.snmmi.org/common/Uploaded%20files/Web/Advocacy%20and%20Initiatives/2023-09-07/NRC%20Comments%20on%20Extravasations%20Rulemaking%209-1-2023.pdf

Footnote 20 (Same as 19)

https://sites.snmmi.org/common/Uploaded%20files/Web/Advocacy%20and%20Initiatives/2023-09-07/NRC%20Comments%20on%20Extravasations%20Rulemaking%209-1-2023.pdf

In the published article, there was an error in the cited statistics and the source associated with Footnote 13. The incorrect citation resulted from human error in our reference management process. Specifically, the wrong link was inadvertently transferred into the final paper from our list of footnote links. Upon further review, we also discovered that the proper link for the 2023 Klick Health study did not contain the original data we referenced. Despite these errors, the main point made in the original paragraph—that consumers are relatively comfortable with AI playing a role in healthcare—remains relevant and important to our discussion. Therefore, the paragraph has been revised accordingly, and a new, accurate footnote has been provided to support our statement.

A correction has been made to **Discussion,** Paragraph Three. This sentence previously stated:

“According to a 2023 study by Klick Health^13^, 79% of U.S. consumers are willing to use AI for their healthcare needs, and 45% believe AI will have a significant impact on healthcare.”

Previous Footnote 13: https://digitalhealthmonitor.org/stateofdigitalhealth23

The corrected sentence appears below:

“According to a recent survey of 2,000 U.S. adults^13^, more Americans trust social media and healthcare websites for advice over a medical professional, 94% trust AI to handle certain health-related tasks, and over half (52%) have consulted large language models like ChatGPT for medical diagnoses, reflecting the growing role of AI in personal healthcare decisions.”

Corrected Footnote 13: https://www.usertesting.com/resources/reports/consumer-perceptions-ai-healthcare

The authors apologize for these errors and state that this does not change the scientific conclusions of the article in any way. The original article has been updated.

