# Supplementary material for: Can ChatGPT help patients understand radiopharmaceutical extravasations?
Source: Front Nucl Med. 2024 Nov 6;4:1469487. doi: 10.3389/fnume.2024.1469487 (PMC11576157; doi:10.3389/fnume.2024.1469487)
Supplement: Supplementary file 1 [file Table1.docx]

**Can ChatGPT Help Patients Understand Radiopharmaceutical Extravasations?**

Madeleine Alvarez^1^

^1^Individual Contributor

*** Correspondence:**Maddie.Alvarez6@gmail.com

**Supplemental File 1.**

**Question 1.**

**What are radiopharmaceutical extravasations?**

**1^st^ ChatGPT response**

Radiopharmaceutical extravasations refer to the accidental leakage of radiopharmaceuticals from a blood vessel into surrounding tissues during intravenous administration. This can occur in nuclear medicine procedures where radioactive substances are injected into the bloodstream to diagnose or treat various diseases. The extravasation of radiopharmaceuticals can potentially lead to several issues:

1. Reduced Diagnostic Accuracy: In diagnostic procedures, extravasation may lead to incorrect imaging results. The leaked substance can cause increased local radiation dose and potentially affect the quality of the diagnostic images.
2. Localized Radiation Dose: The leaked radiopharmaceutical can deliver a higher dose of radiation to the surrounding tissue than intended, potentially causing tissue damage or radiation burns.
3. Altered Pharmacokinetics: Extravasation may change how the radiopharmaceutical is distributed and metabolized in the body, which can affect the effectiveness of the treatment or the accuracy of the diagnostic images.
4. Patient Discomfort and Complications: The extravasation of radiopharmaceuticals can cause discomfort, swelling, and pain at the injection site. In severe cases, it might lead to more serious complications like tissue necrosis.
5. Impact on Treatment Efficacy: In therapeutic procedures, extravasation can lead to reduced efficacy of the treatment, as the intended target tissue may receive a lower dose of the radiopharmaceutical.

Prevention and management of radiopharmaceutical extravasations involve careful injection techniques, monitoring of the injection site, immediate response to any signs of extravasation, and appropriate patient care to minimize the risk and mitigate any effects if extravasation occurs.

References

<https://journals.lww.com/health-physics/Abstract/2022/04000/Radiopharmaceutical_Extravasation__Pragmatic.15.aspx#:~:text=Immediate%20symptoms%20of%20radiopharmaceutical%20extravasation,may%20go%20unnoticed%20until%20later>

<https://jnm.snmjournals.org/content/61/supplement_1/1018#:~:text=Conclusions%3A%20Diagnostic%20radiopharmaceutical%20extravasations%20can,radiopharmaceutical%20extravasations%20also%20cause%20harm>

<https://www.ncbi.nlm.nih.gov/pmc/articles/PMC9512231/#:~:text=Tissue,radiopharmaceutical%20used%2C%20and%20residence%20time>

<https://www.frontiersin.org/articles/10.3389/fnume.2023.1219202/full>

**2^nd^ ChatGPT Response**

Radiopharmaceutical extravasations occur when a radiopharmaceutical—a radioactive compound used in nuclear medicine procedures—leaks or is inadvertently injected into the surrounding tissues instead of the intended vein. This can happen during procedures like positron emission tomography (PET) scans or single-photon emission computed tomography (SPECT) scans, where radiotracers are used to visualize and measure various bodily functions.

The significance of an extravasation can vary based on several factors, such as the type and amount of radiopharmaceutical used, the location of the extravasation, and the patient's overall health. While many extravasations may result in minimal to no harm and resolve on their own, some might cause local tissue damage or affect the quality and accuracy of the imaging due to uneven distribution of the radiotracer.

In clinical settings, it's crucial to minimize the risk of extravasation through careful injection techniques, proper patient assessment, and immediate response if an extravasation is suspected. Monitoring the injection site and educating patients about signs of extravasation are also important steps in managing these incidents.

References

<https://www.versantphysics.com/wp-content/uploads/2020/07/2020-07-04-White-Paper-formatted-1.pdf>

<https://www.ncbi.nlm.nih.gov/pmc/articles/PMC5434120/#:~:text=Extravasation%20of%20therapeutic%20radiopharmaceuticals%20can,immediate%20care%20of%20radiopharmaceutical%20extravasation>

<https://www.frontiersin.org/articles/10.3389/fnume.2023.1219202/full#:~:text=Frontiers%20,agents%2C%20and%20recent%20regulatory%20controversies>

<https://journals.lww.com/health-physics/Abstract/2022/04000/Radiopharmaceutical_Extravasation__Pragmatic.15.aspx#:~:text=Inadvertent%20injection%20of%20a%20radiopharmaceutical,patient%27s%20arm%20and%20skin%20tissue>

**3^rd^ ChatGPT response**

Radiopharmaceutical extravasations refer to the accidental leakage of radiopharmaceuticals into the surrounding tissues during intravenous administration. This event typically occurs in the context of diagnostic or therapeutic nuclear medicine procedures. Radiopharmaceuticals are special drugs containing radioactive material used in these procedures for imaging or treatment purposes.

When a radiopharmaceutical is administered intravenously, it is intended to circulate throughout the body or target specific organs or tissues. However, if the drug leaks out of the vein and into the surrounding tissue, it's known as extravasation. This can happen due to various reasons, such as improper needle placement, fragile veins, or patient movement during injection.

The consequences of radiopharmaceutical extravasations can vary depending on the type and amount of the radiopharmaceutical used and the site of extravasation. Potential issues include:

1. **Altered Imaging Results**: For diagnostic procedures, extravasation can lead to suboptimal images or misleading results, as the concentration of the radiopharmaceutical in the target tissue may be insufficient or uneven.
2. **Radiation Exposure to Unintended Tissues**: Extravasation can result in localized radiation exposure to tissues not intended to receive it, potentially causing tissue damage.
3. **Patient Discomfort or Injury**: Depending on the radiopharmaceutical, extravasation can cause local discomfort, swelling, or more severe tissue injury.

Management of radiopharmaceutical extravasation typically involves symptomatic treatment and monitoring the site of extravasation. In some cases, additional medical interventions might be necessary. It's also essential for nuclear medicine facilities to have protocols in place to minimize the risk of extravasation and to manage it effectively should it occur.

References

<https://www.ncbi.nlm.nih.gov/pmc/articles/PMC5434120/#:~:text=Radiopharmaceutical%20extravasation%20can%20potentially%20lead,interventions%2C%20and%20effectiveness%20of%20these>

<https://pubmed.ncbi.nlm.nih.gov/28303300/#:~:text=Extravasation%20of%20therapeutic%20radiopharmaceuticals%20can,immediate%20care%20of%20radiopharmaceutical%20extravasation>

<https://journals.lww.com/health-physics/Abstract/2022/04000/Radiopharmaceutical_Extravasation__Pragmatic.15.aspx#:~:text=Immediate%20symptoms%20of%20radiopharmaceutical%20extravasation,may%20go%20unnoticed%20until%20later>

<https://pubmed.ncbi.nlm.nih.gov/23835019/#:~:text=Radiopharmaceuticals%20extravasation%20is%20rare%20but,of%20extravasation%20of%20these%20drugs>

<https://journals.lww.com/nuclearmed/Abstract/2021/02000/Effective_Management_of.11.aspx#:~:text=Abstract,octreotate>

**Question 2.**

**How frequently do extravasations occur during radiopharmaceutical administrations?**

**1^st^ ChatGPT response**

Extravasation during radiopharmaceutical administrations is a relatively rare occurrence, but it can vary depending on the type of procedure and the skill of the healthcare professional administering the radiopharmaceutical. In nuclear medicine, extravasation refers to the accidental leakage of a radiopharmaceutical into the tissue surrounding the injection site.

The frequency of extravasations can depend on several factors:

1. Type of Radiopharmaceutical: Some radiopharmaceuticals have a higher viscosity or may be more difficult to administer, potentially increasing the risk of extravasation.
2. Patient Factors: Patient-specific factors such as vein quality, age, and overall health can influence the likelihood of extravasation. For instance, elderly patients or those with fragile veins might be at a higher risk.
3. Technique and Experience: The skill and experience of the healthcare professional performing the injection play a significant role. Proper technique and experience can significantly reduce the risk of extravasation.
4. Volume and Rate of Injection: Larger volumes and faster injection rates may increase the risk of extravasation.

While specific statistics on the frequency of extravasations in radiopharmaceutical administrations might not be readily available, it's generally agreed in the medical community that while the risk exists, it is relatively low with proper technique and precautions. Regular training and adherence to protocols are crucial in minimizing the occurrence of extravasations in nuclear medicine procedures.

References

<https://journals.lww.com/health-physics/Fulltext/2022/11000/Practical_Tools_for_Patient_specific.1.aspx#:~:text=As%20previously%20reported%2C%20diagnostic%20radiopharmaceutical,Currie%20and%20Sanchez%202020>

<https://www.federalregister.gov/documents/2022/12/30/2022-28356/reporting-nuclear-medicine-injection-extravasations-as-medical-events#:~:text=Comments%20Received%3A%20Twenty,3%29%20some%20groups>

<https://www.federalregister.gov/documents/2022/12/30/2022-28356/reporting-nuclear-medicine-injection-extravasations-as-medical-events#:~:text=Comments%20Received%3A%20Twenty,3%29%20some%20groups>

<https://www.ncbi.nlm.nih.gov/pmc/articles/PMC8273265/#:~:text=Adverse%20event%2Fvigilance%20report%20database%20reviews,regulatory%20exemption%20for%20required%20reporting>

**2^nd^ ChatGPT Response**

Extravasation during radiopharmaceutical administrations is a recognized issue in nuclear medicine. Reports indicate that radiopharmaceutical extravasation (RPE) occurs quite frequently, with 37 publications documenting 3016 cases of diagnostic radiopharmaceutical extravasation. However, there is a noted scarcity of evidence on the clinical consequences of these events. The frequency of extravasation during radiopharmaceutical injections is generally reported to be more than 10%, a figure that underscores the regularity with which this issue occurs in clinical settings. Another study specifically mentions a frequency of 15% within diagnostic nuclear medicine imaging, highlighting the variability due to center- and individual-specific factors.

These figures underscore the importance of awareness and management strategies for extravasation in the administration of radiopharmaceuticals to minimize potential risks and ensure accurate dosimetry.

References

<https://www.researchgate.net/journal/European-Journal-of-Nuclear-Medicine-and-Molecular-Imaging-1619-7089/publication/315327059_Consequences_of_radiopharmaceutical_extravasation_and_therapeutic_interventions_a_systematic_review/links/5f744e63a6fdcc0086488a11/Consequences-of-radiopharmaceutical-extravasation-and-therapeutic-interventions-a-systematic-review.pdf#:~:text=Radiopharmaceutical%20extravasation%20,published%20reports%20of%20radiopharmaceutical%20extravasations>

<https://journals.lww.com/health-physics/Fulltext/2022/11000/Practical_Tools_for_Patient_specific.1.aspx#:~:text=As%20previously%20reported%2C%20diagnostic%20radiopharmaceutical,Currie%20and%20Sanchez%202020>

<https://pubmed.ncbi.nlm.nih.gov/35838538/#:~:text=Extravasation%20during%20radiopharmaceutical%20injection%20may,free%20and%20publicly%20av%20%E2%80%A6>

<https://www.frontiersin.org/articles/10.3389/fnume.2023.1171918/full#:~:text=The%20frequency%20of%20extravasation%20within,entering%20systemic%20circulation%20as%20intended>

**3^rd^ ChatGPT response**

Extravasation during radiopharmaceutical administration is a known complication, though it is relatively rare. The frequency of extravasation varies depending on several factors, including the type of radiopharmaceutical used, the technique of administration, and patient-specific factors.

In general, extravasation occurs in a small percentage of nuclear medicine procedures. For instance, studies have reported extravasation rates ranging from less than 1% to around 5% in various settings. However, it's important to note that these rates can vary widely based on the specific context and the population being studied.

In nuclear medicine, extravasation can impact both the diagnostic quality of the imaging study and, in therapeutic settings, the efficacy of the treatment. It can also potentially lead to localized radiation damage, though this is rare with diagnostic doses. To minimize the risk of extravasation, proper injection techniques and careful patient monitoring are essential. If extravasation is suspected, immediate actions are taken to mitigate any potential harm.

It's also worth noting that the ongoing advancements in injection techniques and the increasing awareness among healthcare professionals about extravasation risks may contribute to a decrease in its occurrence over time.

References

<https://pubmed.ncbi.nlm.nih.gov/33443961/>

<https://jnm.snmjournals.org/content/early/2023/06/29/jnumed.123.265717>

<https://lucerno.com/extravasation-in-nuclear-medicine/>

**Question 3.**

**How should clinicians address a suspected radiopharmaceutical extravasation?**

**1^st^ ChatGPT response**

When clinicians suspect radiopharmaceutical extravasation, which is the accidental leakage of a radiopharmaceutical into the surrounding tissues during an injection, they should follow a set of recommended steps to minimize any potential harm and manage the situation effectively:

1. Immediate Response:
   - Stop the Injection: Immediately stop the radiopharmaceutical injection once extravasation is suspected.
   - Mark the Site: Mark the extravasation area with a semi-permanent marker to help in future assessments and interventions.
2. Minimize Spread:
   - Elevate the Limb: Elevating the affected limb can help reduce the spread of the radiopharmaceutical.
   - Apply Cold Packs: Initially, cold packs can be applied to the affected area to constrict blood vessels and reduce the spread. However, in some cases, depending on the type of radiopharmaceutical, warm packs might be more appropriate.
3. Assessment and Documentation:
   - Visual Assessment: Observe the site for swelling, redness, or any other signs of tissue damage.
   - Document the Event: It is important to document the extravasation incident, including the time, volume of extravasation, and the actions taken.
4. Patient Monitoring and Follow-up:
   - Monitor the Patient: Continuous monitoring for any signs of adverse effects or tissue damage is crucial.
   - Follow-Up Imaging: In some cases, imaging of the affected area may be necessary to assess the extent of radiopharmaceutical spread and tissue damage.
5. Consultation and Reporting:
   - Consult a Specialist: Consultation with a nuclear medicine specialist or a radiation safety officer is recommended for guidance on further management.
   - Report the Incident: Report the extravasation to the relevant authorities or departments within the healthcare facility as per institutional protocols.
6. Patient Education and Reassurance:
   - Educate the Patient: Inform the patient about the extravasation, potential effects, and the steps taken to manage it.
   - Reassure the Patient: Reassure the patient, as most extravasations result in minimal or no long-term harm.
7. Review and Preventive Measures:
   - Review Injection Techniques: Review and reinforce proper injection techniques among staff to prevent future incidents.
   - Update Protocols: Consider updating institutional protocols and training based on the incident to enhance safety.

Each case of radiopharmaceutical extravasation might require a specific approach based on the type of radiopharmaceutical used, the volume extravasated, and the patient's overall condition. It's important for clinicians to be familiar with the properties of the radiopharmaceuticals they use and the best practices for managing extravasation specific to those substances.

References

<https://pubmed.ncbi.nlm.nih.gov/23835019/>

<https://www.frontiersin.org/articles/10.3389/fnume.2023.1244660/full>

**2^nd^ ChatGPT Response**

When clinicians suspect radiopharmaceutical extravasation (the leakage of a radiopharmaceutical into the surrounding tissue during injection), they should follow a series of steps to manage and mitigate the situation effectively:

1. **Stop Injection Immediately**: As soon as extravasation is suspected, stop the administration of the radiopharmaceutical to prevent further leakage.
2. **Mild Pressure Application**: Apply mild pressure to the site of injection to help contain the spread of the radiopharmaceutical. Avoid excessive pressure, which can cause further tissue damage.
3. **Elevate the Affected Limb**: Elevating the limb can reduce swelling and help disperse any radiopharmaceutical that has leaked.
4. **Cold/Hot Packs**: Initially, apply a cold pack to the affected area to constrict blood vessels and minimize the spread. After a few hours, some protocols recommend switching to a warm pack to enhance dispersion and absorption of the radiopharmaceutical.
5. **Document the Incident**: Record the extent of the extravasation, the estimated volume of leakage, the time, and the actions taken. This information is crucial for future treatment and follow-up.
6. **Symptom Monitoring**: Watch for signs of inflammation or tissue damage. Symptoms might include redness, swelling, and pain.
7. **Follow-Up Imaging**: Depending on the radiopharmaceutical used, additional imaging may be required to assess the extent of the spread and to plan for any necessary treatment.
8. **Inform the Patient**: Explain what has happened to the patient, the potential effects, and what to watch for in terms of symptoms or complications.
9. **Consult a Specialist**: In some cases, especially with high doses or specific agents, consult a radiologist, nuclear medicine physician, or a radiation safety officer for further guidance.
10. **Review and Learn**: After the incident, review the procedure to understand how the extravasation occurred and how similar incidents can be prevented in the future.

It's important to follow the specific guidelines and protocols of the healthcare facility and the nature of the radiopharmaceutical involved. Different institutions may have slightly different procedures, and some situations may require specific actions depending on the radiopharmaceutical's properties and the amount that was extravasated.

References

<https://pubmed.ncbi.nlm.nih.gov/23835019/>

<https://pubmed.ncbi.nlm.nih.gov/28303300/>

**3^rd^ ChatGPT response**

Radiopharmaceutical extravasation, which is the leakage of a radiopharmaceutical into the surrounding tissue during an intravenous injection, should be managed carefully by clinicians due to its potential to cause localized radiation exposure and possibly affect the quality of diagnostic imaging. Here's a general approach to addressing a suspected radiopharmaceutical extravasation:

1. **Immediate Actions**:
   - Stop the injection immediately upon suspicion of extravasation.
   - Leave the needle or catheter in place initially.
   - Elevate the affected limb to reduce swelling.
2. **Assessment and Documentation**:
   - Carefully assess the extent of extravasation.
   - Document the amount of radiopharmaceutical extravasated, the time of occurrence, and the patient’s symptoms.
3. **Minimizing Spread**:
   - Gently apply pressure (not too firm to avoid further tissue damage) around the site to help disperse the radiopharmaceutical.
   - Consider applying a cold pack initially to constrict blood vessels and reduce the spread. After some time, a warm pack can help in dispersing the radiopharmaceutical.
4. **Symptom Management**:
   - Monitor the patient for any signs of inflammation or tissue damage.
   - Provide pain relief if necessary.
5. **Follow-up and Imaging Considerations**:
   - Consider the potential impact on image quality. Inform the nuclear medicine physician or radiologist about the extravasation, as it may affect the interpretation of the scan.
   - Follow-up with the patient to monitor for any delayed symptoms or complications.
6. **Safety and Preventive Measures**:
   - Review the extravasation protocol and techniques to prevent future occurrences.
   - Train staff on proper injection techniques and the handling of radiopharmaceuticals.
7. **Reporting**:
   - Report the incident according to institutional policies and possibly to regulatory authorities, depending on the severity and the amount of radiopharmaceutical involved.

It's important to note that the specific actions and protocols might vary depending on the type of radiopharmaceutical used, the amount of extravasation, and the patient's overall health condition. Clinicians should always refer to their institution’s guidelines and collaborate with the nuclear medicine department for the best patient care.

References

<https://www.frontiersin.org/articles/10.3389/fnume.2023.1219202/full>

<https://jnm.snmjournals.org/content/64/3/491>

<https://pubmed.ncbi.nlm.nih.gov/23835019/>

**Question 4.**

**How important is it to mitigate a radiopharmaceutical extravasation quickly?**

**1^st^ ChatGPT response**

Mitigating a radiopharmaceutical extravasation quickly is quite important for several reasons:

1. **Tissue Damage**: Radiopharmaceuticals, due to their radioactive nature, can cause significant tissue damage at the site of extravasation. Quick action can minimize this damage.
2. **Accurate Diagnostic Imaging**: If the radiopharmaceutical is being used for diagnostic imaging (like a PET scan), an extravasation can lead to poor image quality and misinterpretation. Rapid response helps ensure the accuracy of diagnostic results.
3. **Radiation Exposure**: Quick mitigation limits the duration of radiation exposure to the surrounding tissue and reduces the risk of long-term effects like radiation-induced necrosis.
4. **Patient Comfort and Safety**: Rapid intervention can alleviate patient discomfort and prevent more serious complications, contributing to overall patient safety and care quality.
5. **Compliance with Safety Protocols**: Promptly addressing extravasation is in line with safety protocols and standards in nuclear medicine, reflecting good clinical practice.

Overall, the quick mitigation of a radiopharmaceutical extravasation is critical to ensure patient safety, the integrity of diagnostic processes, and the minimization of potential tissue damage.

References

<https://pubmed.ncbi.nlm.nih.gov/28303300/>

<https://www.frontiersin.org/articles/10.3389/fnume.2023.1127692/full#:~:text=Radiopharmaceutical%20extravasations%20provide%20zero%20patient,transparency%20exists%20regarding%20these%20extravasations>

**2^nd^ ChatGPT Response**

Mitigating a radiopharmaceutical extravasation, or the leakage of radioactive material into surrounding tissues during injection, is quite important for several reasons:

1. **Tissue Damage**: Radiopharmaceuticals are designed to emit radiation, which can be harmful to tissues if not properly contained. Extravasation can lead to localized radiation doses significantly higher than intended, potentially causing tissue damage or necrosis.
2. **Diagnostic Accuracy**: In diagnostic procedures, such as PET scans, the accuracy of the images relies on the predictable distribution of the radiotracer. Extravasation can lead to misleading results, as the concentration of the radiotracer in the extravasation area might be misinterpreted as a site of disease.
3. **Therapeutic Effectiveness**: For therapeutic radiopharmaceuticals, it's crucial that the intended dose reaches the target tissue. Extravasation can reduce the effective dose delivered to the disease site, diminishing treatment efficacy.
4. **Patient Comfort and Safety**: Extravasation can cause pain, swelling, and discomfort for the patient. In severe cases, it may require surgical intervention to remove necrotic tissue or address severe damage.

Quick mitigation efforts typically include:

- - **Stopping the injection** immediately upon detection of extravasation.
  - **Applying pressure** to the site (without massaging) to help contain the spread.
  - **Elevating the affected limb** to reduce swelling.
  - **Monitoring** the site for signs of tissue damage and following up with appropriate medical care, possibly including imaging to assess the extent of the spread.

Each facility will have protocols in place to deal with such events, emphasizing the importance of training and awareness among staff handling radiopharmaceuticals. So, quick and effective mitigation of radiopharmaceutical extravasation is critical to ensure patient safety, the accuracy of diagnostic tests, and the effectiveness of therapeutic treatments.

References

<https://www.ncbi.nlm.nih.gov/pmc/articles/PMC5434120/#:~:text=Extravasation%20of%20therapeutic%20radiopharmaceuticals%20can,immediate%20care%20of%20radiopharmaceutical%20extravasation>

<https://journals.lww.com/health-physics/Abstract/2022/04000/Radiopharmaceutical_Extravasation__Pragmatic.15.aspx#:~:text=Inadvertent%20injection%20of%20a%20radiopharmaceutical,patient%27s%20arm%20and%20skin%20tissue>

<https://ejnmmiphys.springeropen.com/articles/10.1186/s40658-021-00378-3#:~:text=Background%20Extravasation%20of%20radiopharmaceuticals%20used,This>

<https://jnm.snmjournals.org/content/61/supplement_1/1018#:~:text=1018%20Objectives%3A%20Effects%20of%20therapeutic,true%20extravasation%20frequency%2C%20insufficient>

**3^rd^ ChatGPT response**

Mitigating a radiopharmaceutical extravasation, which is the accidental injection of a radiopharmaceutical into tissue or space outside of the intended vein, is quite important for several reasons:

1. **Reduced Diagnostic Accuracy**: Extravasation can lead to suboptimal distribution of the radiopharmaceutical, potentially compromising the diagnostic accuracy of nuclear medicine imaging.
2. **Tissue Damage**: Some radiopharmaceuticals, depending on their radiative properties and chemical nature, can cause tissue damage at the site of extravasation. This can lead to inflammation, necrosis, or other local tissue reactions.
3. **Radiation Exposure**: Localized concentration of the radiopharmaceutical at the extravasation site can lead to increased radiation dose to the affected tissue. This is particularly concerning with radiopharmaceuticals that emit beta or alpha particles, which have a high linear energy transfer and can cause significant local tissue damage.
4. **Patient Comfort and Anxiety**: Extravasation can cause discomfort or pain to the patient, and knowing that a radiopharmaceutical has not been administered correctly can increase a patient's anxiety.
5. **Need for Additional Interventions**: If extravasation occurs, additional interventions might be necessary, such as localized cooling, elevation of the affected limb, or in more severe cases, surgical intervention.
6. **Regulatory and Safety Concerns**: Proper administration of radiopharmaceuticals is a key aspect of nuclear medicine practice. Extravasation incidents may need to be reported to regulatory authorities, and repeated incidents can indicate a need for improved training or changes in procedure.

Due to these reasons, quick identification and mitigation of a radiopharmaceutical extravasation are important to ensure patient safety, diagnostic accuracy, and adherence to radiation safety principles. The specific response to extravasation can vary depending on the radiopharmaceutical involved, the amount extravasated, and the patient's condition.

References

<https://www.ncbi.nlm.nih.gov/pmc/articles/PMC5434120/#:~:text=Extravasation%20of%20therapeutic%20radiopharmaceuticals%20can,immediate%20care%20of%20radiopharmaceutical%20extravasation>

<https://journals.lww.com/health-physics/Abstract/2022/04000/Radiopharmaceutical_Extravasation__Pragmatic.15.aspx#:~:text=Inadvertent%20injection%20of%20a%20radiopharmaceutical,patient%27s%20arm%20and%20skin%20tissue>

<https://ejnmmiphys.springeropen.com/articles/10.1186/s40658-021-00378-3#:~:text=Background%20Extravasation%20of%20radiopharmaceuticals%20used,This>

<https://jnm.snmjournals.org/content/61/supplement_1/1018#:~:text=1018%20Objectives%3A%20Effects%20of%20therapeutic,true%20extravasation%20frequency%2C%20insufficient>

**Question 5.**

**Who should receive a report of a radiopharmaceutical extravasation?**

**1^st^ ChatGPT response**

In the case of a radiopharmaceutical extravasation, it's important to follow established protocols and report the incident to the appropriate parties. Typically, these would include:

1. **Nuclear Medicine Physician or Radiologist**: The supervising physician should be informed immediately. They can assess the severity of the extravasation and provide guidance on further steps.
2. **Radiation Safety Officer (RSO)**: The RSO should be notified as they are responsible for managing radiation safety issues within the facility. They will document the incident and may provide input on mitigating any potential radiation risks.
3. **Patient's Referring Physician**: Informing the patient’s primary care or referring physician is crucial as they need to be aware of any potential complications or changes in the patient’s condition.
4. **Nuclear Medicine Technologist or Staff Involved in the Procedure**: They should be aware of the incident as it may affect the interpretation of the scan and to improve future practices.
5. **Hospital or Clinic Administration**: Depending on the severity and the policies of the institution, the administration may need to be informed for record-keeping and quality control purposes.
6. **Regulatory Bodies**: In some cases, especially if there is a significant radiation dose involved or if it leads to severe patient outcomes, it may be necessary to report the incident to relevant regulatory bodies or health authorities.
7. **Patient**: It's important to inform the patient about the extravasation, potential effects, and any follow-up actions that might be necessary.

Each facility may have specific protocols and reporting chains for such incidents, so it's important to follow the guidelines and procedures in place at your institution.

References

No reference

**2^nd^ ChatGPT Response**

When a radiopharmaceutical extravasation occurs, it's important to report it promptly and to the appropriate parties to ensure patient safety and proper incident handling. Here are the key individuals and entities who should typically receive a report of a radiopharmaceutical extravasation:

1. **Nuclear Medicine Technologist/Attending Radiologist:** The primary individuals performing or overseeing the procedure should be immediately informed as they are responsible for initial assessment and immediate care.
2. **Patient's Physician:** The physician in charge of the patient's care should be notified to understand the potential implications of the extravasation and to monitor for any adverse effects.
3. **Radiation Safety Officer (RSO):** The RSO should be informed as they are responsible for overseeing radiation safety protocols and can provide guidance on managing and documenting the incident.
4. **Hospital or Clinic Administration:** Depending on the institution's policies, the administration may need to be informed for incident reporting purposes and to follow up on any required procedural changes.
5. **Regulatory Bodies:** In some regions, certain types of radiopharmaceutical incidents must be reported to regulatory authorities or health departments to comply with national or local regulations.
6. **Risk Management/Legal Department:** To ensure all legal and regulatory guidelines are followed, and to help manage any potential liability or regulatory issues.

It's crucial to follow the specific protocols and procedures of your workplace and adhere to any local laws and regulations regarding radiopharmaceuticals and radiation safety. Each facility might have its own specific protocol for such events, including documentation and follow-up procedures. Always consult with your facility's policies and senior staff when handling such incidents.

References

No references

**3^rd^ ChatGPT response**

A report of a radiopharmaceutical extravasation should be communicated to several key parties:

1. **Nuclear Medicine Physician or Radiologist**: The primary physician overseeing the nuclear medicine procedure should be informed immediately. They can assess the extent of extravasation and decide on further action.
2. **Referring Physician**: The patient's referring physician should be notified, especially if the extravasation might impact the patient's overall treatment plan or health status.
3. **Radiation Safety Officer (RSO) or Nuclear Medicine Safety Committee**: In many institutions, the RSO or a dedicated safety committee oversees incidents involving radiopharmaceuticals. Reporting the extravasation to them ensures that safety protocols are followed and any necessary actions to prevent future incidents are taken.
4. **Patient**: It is important to inform the patient about the extravasation, including potential effects and any necessary follow-up care or monitoring.
5. **Medical Records**: Documenting the extravasation in the patient's medical records is crucial for future reference and ongoing care.
6. **Regulatory Authorities (if required)**: In certain cases, especially if the extravasation is severe or leads to significant adverse effects, it may be necessary to report the incident to regulatory authorities in accordance with national or regional regulations.

Each institution may have specific protocols for handling and reporting radiopharmaceutical extravasations, so it's important to be familiar with and follow these procedures.

References

No references

**Question 6.**

**Can radiopharmaceutical extravasations be prevented or minimized?**

**1^st^ ChatGPT response**

Radiopharmaceutical extravasations, which occur when radiopharmaceuticals inadvertently leak into the surrounding tissues during intravenous administration, can be minimized or prevented through several measures:

1. **Proper Injection Technique**: Using the correct technique for intravenous injections is crucial. This includes selecting the appropriate vein, ensuring proper needle placement, and confirming venous access before administering the radiopharmaceutical.
2. **Patient Education**: Informing the patient about the procedure and advising them to report any discomfort or pain during the injection can help in early detection of extravasation.
3. **Use of Small Veins**: Avoiding the use of small or fragile veins can reduce the risk of extravasation. Larger, more stable veins are preferable.
4. **Monitoring During Injection**: Close monitoring of the injection site during and immediately after the injection can help in early detection of any leakage.
5. **Slow Injection Rate**: Administering the radiopharmaceutical slowly and steadily can minimize the pressure on the vein and reduce the risk of extravasation.
6. **Use of Butterfly Needles**: Some practitioners recommend using butterfly needles for better control and stability during the injection.
7. **Post-Injection Care**: After the injection, observing the site for any signs of swelling, redness, or leakage is important. Immediate action should be taken if extravasation is suspected.
8. **Training and Experience**: Ensuring that personnel administering radiopharmaceuticals are well-trained and experienced can significantly reduce the risk of extravasation.
9. **Equipment Check**: Regular checks and maintenance of injection equipment can prevent malfunctions that might lead to extravasation.

By following these guidelines, the risk of radiopharmaceutical extravasation can be significantly reduced, although it may not be completely eliminated. In cases where extravasation does occur, prompt and appropriate management is essential to minimize any potential harm.

References

<https://pubmed.ncbi.nlm.nih.gov/23835019/>

<https://www.frontiersin.org/articles/10.3389/fnume.2023.1219202/full>

<https://altuslearn.com/radiopharmaceutical-extravasations-hazards-mitigation-and-prevention/>

**2^nd^ ChatGPT Response**

Radiopharmaceutical extravasation, which refers to the accidental leakage of radiopharmaceuticals into surrounding tissues during administration, can be a concern in nuclear medicine procedures. To prevent or minimize this issue, several strategies can be employed:

1. **Proper Injection Technique**: Ensuring the correct technique for intravenous injection is vital. This includes verifying the proper placement of the needle or catheter in the vein before administering the radiopharmaceutical.
2. **Patient Education**: Informing the patient about the procedure and instructing them to report any discomfort or pain during the injection can help in early detection of extravasation.
3. **Use of Small Gauge Needles**: Using smaller gauge needles can reduce the risk of damaging the vein and causing extravasation.
4. **Slow Injection Rate**: Administering the radiopharmaceutical slowly and carefully can reduce the risk of pressure buildup and leakage.
5. **Monitoring Injection Site**: Careful monitoring of the injection site for signs of swelling, redness, or pain can help in early detection of extravasation.
6. **Post-Injection Care**: After the injection, applying pressure to the site (without massaging) and observing it for a short period can help in detecting any immediate issues.
7. **Training and Experience**: Ensuring that the personnel administering the radiopharmaceuticals are well-trained and experienced can significantly reduce the risk of extravasation.
8. **Equipment Check**: Regular maintenance and checks of the equipment used for injections can also help in preventing issues related to faulty administration devices.
9. **Patient Selection and Assessment**: Assessing the patient's venous access and overall condition prior to the procedure can guide the choice of the most appropriate injection site and technique.
10. **Emergency Protocols**: Having protocols in place for managing extravasation if it occurs, including the use of specific interventions to limit tissue exposure to the radiopharmaceutical, can minimize potential harm.

While it may not be possible to completely eliminate the risk of radiopharmaceutical extravasation, these measures can significantly reduce its likelihood and impact.

References

<https://pubmed.ncbi.nlm.nih.gov/23835019/>

<https://www.frontiersin.org/articles/10.3389/fnume.2023.1219202/full>

**3^rd^ ChatGPT response**

Radiopharmaceutical extravasation, which refers to the accidental leakage of radiopharmaceuticals into the surrounding tissue during intravenous administration, can be minimized or prevented through various measures:

1. **Proper Injection Technique**: Using the correct technique for intravenous injections is crucial. This includes verifying the patency of the vein both before and after injection.
2. **Use of Small Gauge Needles**: Smaller gauge needles can reduce the risk of damaging the vein and causing extravasation.
3. **Patient Positioning**: Ensuring the patient is in a comfortable and correct position can reduce the movement during the procedure, lowering the risk of extravasation.
4. **Slow Injection Rate**: Administering the radiopharmaceutical slowly and steadily can minimize the risk of overwhelming the vein and causing leakage.
5. **Frequent Monitoring**: Constant monitoring during the injection process helps in immediately identifying any signs of extravasation.
6. **Education and Training**: Healthcare professionals should be well-trained and educated in the techniques and risks associated with radiopharmaceutical injections.
7. **Use of Appropriate Veins**: Selecting the appropriate vein for injection, usually a vein with good blood flow and away from joints, can reduce the risk.
8. **Patient Education**: Informing patients about the importance of staying still and reporting any discomfort during the procedure can aid in early detection of problems.
9. **Prompt Response to Extravasation Signs**: If extravasation is suspected, immediate action should be taken, such as stopping the injection and applying appropriate interventions.
10. **Use of Tourniquets**: Careful use of tourniquets can help control the vein's blood flow, but they should be used judiciously to avoid additional complications.

It's important to note that while these measures can significantly reduce the risk, they may not completely eliminate the possibility of radiopharmaceutical extravasation. Each situation should be evaluated individually, and healthcare providers should be prepared to manage extravasation if it occurs.

References

<https://pubmed.ncbi.nlm.nih.gov/23835019/>

<https://altuslearn.com/radiopharmaceutical-extravasations-hazards-mitigation-and-prevention/>

**Question 7.**

**Should a patient be concerned about a diagnostic radiopharmaceutical extravasation?**

**1^st^ ChatGPT response**

Extravasation of a diagnostic radiopharmaceutical can be a concern for patients undergoing nuclear medicine procedures. While most radiopharmaceuticals used for diagnostic purposes have relatively low radiation doses and short half-lives, the local tissue reaction and the potential for altered diagnostic study results are the primary concerns. Here are some key points to consider:

1. **Local Tissue Reaction**: Extravasation of a radiopharmaceutical can cause local tissue irritation or damage, depending on the volume and the type of radiopharmaceutical extravasated. However, most diagnostic radiopharmaceuticals are designed to minimize tissue irritation.
2. **Altered Diagnostic Results**: Extravasation may lead to suboptimal diagnostic images. This is because the intended distribution of the radiopharmaceutical throughout the body is altered, potentially leading to misinterpretation of the nuclear medicine scan.
3. **Radiation Dose**: Although the radiation dose from diagnostic radiopharmaceuticals is generally low, local radiation dose to the extravasation site can be higher than intended. This is typically not a significant health risk but should be monitored.
4. **Management and Monitoring**: In case of extravasation, the site is usually monitored for any adverse reactions. Cooling or warming the area and elevating the affected limb can help mitigate the effects. It's important to inform the nuclear medicine personnel immediately if an extravasation is suspected.
5. **Rare Complications**: Severe complications from extravasations of diagnostic radiopharmaceuticals are rare but can occur. It's important for patients to be aware of the possibility and to discuss any concerns with their healthcare provider.

In summary, while extravasation of a diagnostic radiopharmaceutical can be a concern, it is generally not associated with severe health risks. The primary issues are potential local tissue reactions and the impact on the quality of the diagnostic images. Patients should be informed about the possibility of extravasation and the steps to be taken if it occurs.

References

<https://www.frontiersin.org/articles/10.3389/fnume.2023.1219202/full#B7>

<https://pubmed.ncbi.nlm.nih.gov/35085120/>

**2^nd^ ChatGPT Response**

In medical imaging, radiopharmaceutical extravasation refers to the accidental leaking of a radiopharmaceutical into the surrounding tissue during an injection for a diagnostic imaging procedure, such as a PET scan or a nuclear medicine study. While this can happen occasionally, the degree of concern for a patient depends on several factors:

1. **Type and Amount of Radiopharmaceutical**: Some radiopharmaceuticals are more reactive or toxic to tissues than others. The amount of the substance that extravasates can also influence the potential for tissue damage.
2. **Patient's Health and Medical History**: Patients with certain pre-existing conditions or sensitivities may be more at risk for complications from extravasation.
3. **Location of Extravasation**: Certain areas of the body may be more sensitive to the effects of extravasation, potentially leading to more significant complications.
4. **Immediate Response**: Prompt and appropriate management of extravasation can reduce the risk of serious complications. This often includes measures to limit the spread of the radiopharmaceutical and to mitigate its effects on the surrounding tissue.

In general, while radiopharmaceutical extravasation is not desirable, most instances are managed effectively without serious long-term consequences for the patient. However, it's important for any concerns to be addressed with the healthcare team involved in the procedure. They can provide specific information related to the type of radiopharmaceutical used, the expected outcomes, and any necessary follow-up care.

References

<https://www.versantphysics.com/wp-content/uploads/2020/07/2020-07-04-White-Paper-formatted-1.pdf>

<https://www.ncbi.nlm.nih.gov/pmc/articles/PMC5434120/#:~:text=Extravasation%20of%20therapeutic%20radiopharmaceuticals%20can,immediate%20care%20of%20radiopharmaceutical%20extravasation>

<https://www.frontiersin.org/articles/10.3389/fnume.2023.1219202/full#:~:text=Frontiers%20,agents%2C%20and%20recent%20regulatory%20controversies>

<https://journals.lww.com/health-physics/Abstract/2022/04000/Radiopharmaceutical_Extravasation__Pragmatic.15.aspx#:~:text=Inadvertent%20injection%20of%20a%20radiopharmaceutical,patient%27s%20arm%20and%20skin%20tissue>

**3^rd^ ChatGPT response**

When it comes to diagnostic radiopharmaceutical extravasation, which is the accidental leakage of a radiopharmaceutical into surrounding tissues during an injection for a diagnostic imaging procedure, there are several factors to consider:

1. **Potential for Tissue Damage**: Radiopharmaceutical extravasation can potentially lead to tissue damage, especially in the case of therapeutic radiopharmaceuticals, which can cause severe soft tissue lesions.
2. **Frequency and Severity**: These incidents are rare, but they may have serious clinical implications. However, there is a lack of comprehensive data on the incidence, medical consequences, and effective interventions for such events.
3. **Immediate Symptoms**: Immediate symptoms of extravasation may include swelling, edema, pain, or numbness at the site of the extravasation, along with inflammation and possible drainage from the site.
4. **Impact on Diagnosis and Treatment**: Diagnostic radiopharmaceutical extravasations can potentially affect patient management and expose tissue to higher doses than anticipated, which can have implications for both diagnosis and treatment.

Given these considerations, a patient should be aware of the potential risks associated with radiopharmaceutical extravasation. While the occurrence is rare and often managed effectively without long-term consequences, it is important for patients to discuss any concerns with their healthcare provider. This discussion can provide specific information related to the type of radiopharmaceutical used, potential outcomes, and any necessary follow-up care. Prompt recognition and appropriate management of extravasation can significantly reduce the risk of serious complications.

References

<https://www.versantphysics.com/wp-content/uploads/2020/07/2020-07-04-White-Paper-formatted-1.pdf>

<https://www.ncbi.nlm.nih.gov/pmc/articles/PMC5434120/#:~:text=Extravasation%20of%20therapeutic%20radiopharmaceuticals%20can,immediate%20care%20of%20radiopharmaceutical%20extravasation>

<https://www.frontiersin.org/articles/10.3389/fnume.2023.1219202/full#:~:text=Frontiers%20,agents%2C%20and%20recent%20regulatory%20controversies>

<https://journals.lww.com/health-physics/Abstract/2022/04000/Radiopharmaceutical_Extravasation__Pragmatic.15.aspx#:~:text=Inadvertent%20injection%20of%20a%20radiopharmaceutical,patient%27s%20arm%20and%20skin%20tissue>

**Question 8.**

**How does a radiopharmaceutical extravasation affect a patient?**

**1^st^ ChatGPT response**

Radiopharmaceutical extravasation, which occurs when a radiopharmaceutical agent is inadvertently injected into the tissue surrounding a vein rather than directly into the vein itself, can have various effects on a patient, depending on several factors like the type of radiopharmaceutical used, the amount of extravasation, and the patient's overall health condition. Here are some of the potential effects:

1. **Local Tissue Reaction**: The most common effect is a local tissue reaction. This can range from mild irritation and inflammation to more severe tissue damage. Some radiopharmaceuticals are more irritating to tissues than others.
2. **Altered Imaging Results**: Extravasation can affect the quality and accuracy of the diagnostic imaging for which the radiopharmaceutical was intended. It can lead to false or misleading results, as the concentration of the agent in the extravasated area may be misinterpreted as a pathological finding.
3. **Pain and Discomfort**: The patient may experience pain or discomfort at the injection site. This is usually temporary but can be distressing.
4. **Radiation Exposure**: In cases of significant extravasation, there can be a concern about localized radiation exposure to the tissue. This is more of a concern with therapeutic radiopharmaceuticals, which carry higher levels of radioactivity compared to diagnostic agents.
5. **Infection and Necrosis**: In rare cases, particularly if the extravasation is not promptly addressed, there can be a risk of infection or tissue necrosis (death of tissue).
6. **Psychological Impact**: The incident itself can cause anxiety or distress to the patient, particularly if it leads to additional procedures or interventions.
7. **Delayed or Repeat Procedures**: If the imaging study is compromised due to extravasation, it may need to be repeated, leading to delays in diagnosis and treatment, as well as additional radiation exposure.

Management of radiopharmaceutical extravasation typically involves immediate measures such as elevating the affected limb, applying a cold compress to reduce inflammation, and monitoring the site for any adverse reactions. In most cases, the effects are minor and resolve without serious complications. However, it's important for healthcare providers to take steps to minimize the risk of extravasation and to manage it effectively if it occurs.

References

<https://pubmed.ncbi.nlm.nih.gov/28303300/#:~:text=Extravasation%20of%20therapeutic%20radiopharmaceuticals%20can,immediate%20care%20of%20radiopharmaceutical%20extravasation>

<https://www.frontiersin.org/articles/10.3389/fmed.2021.684157/full#:~:text=Although%20an%20extravasation%20is%20typically,ionizing%20radiation%20on%20patient%20tissue>

<https://pubmed.ncbi.nlm.nih.gov/35085120/#:~:text=Immediate%20symptoms%20of%20radiopharmaceutical%20extravasation,may%20go%20unnoticed%20until%20later>

<https://jnm.snmjournals.org/content/64/3/485#:~:text=The%20deleterious%20effects%20of%20high,on%20clinical%20adverse%20events>

**2^nd^ ChatGPT Response**

Radiopharmaceutical extravasation is an incident that occurs when a radiopharmaceutical—a radioactive substance used in nuclear medicine imaging—leaks out of the vein and into the surrounding tissues during intravenous administration. Its effects on a patient can vary depending on several factors including the type of radiopharmaceutical used, the amount of leakage, and the location of the extravasation. Here are some potential effects:

1. **Localized Radiation Injury:** The primary concern with extravasation of radiopharmaceuticals is the localized radiation dose to the tissue surrounding the injection site. This can lead to tissue damage and necrosis in severe cases, especially with high-dose or beta-emitting radiopharmaceuticals.
2. **Altered Diagnostic Accuracy:** Extravasation can affect the accuracy of the diagnostic scan. Since the radiopharmaceutical is not fully available for uptake by the target organ or tissue, the resulting images may be misleading, showing reduced uptake or unexpected distribution patterns, which can lead to misdiagnosis or the need for repeat imaging.
3. **Pain and Swelling:** The patient may experience pain, swelling, or discomfort at the injection site. This is typically mild and temporary but can be more severe depending on the volume and type of radiopharmaceutical extravasated.
4. **Delayed Tissue Healing:** The radiation dose delivered to the tissues may delay healing or cause local skin changes, potentially leading to longer-term cosmetic or functional issues.
5. **Psychological Impact:** Patients may experience anxiety or distress, especially if they are informed about the extravasation and its potential consequences.

In cases of extravasation, immediate action is typically taken to minimize the dose to the affected tissue, such as applying a cold pack to reduce local blood flow or elevating the limb to decrease swelling. The severity of the effects largely depends on the specific circumstances of the extravasation, including the radioisotope used and the patient’s overall health. Monitoring and follow-up care are important to manage any potential complications. Medical professionals handling radiopharmaceuticals are trained to minimize the risk of extravasation and to manage it effectively if it occurs.

References

<https://www.ncbi.nlm.nih.gov/pmc/articles/PMC5434120/#:~:text=Extravasation%20of%20therapeutic%20radiopharmaceuticals%20can,immediate%20care%20of%20radiopharmaceutical%20extravasation>

<https://journals.lww.com/health-physics/Abstract/2022/04000/Radiopharmaceutical_Extravasation__Pragmatic.15.aspx#:~:text=Immediate%20symptoms%20of%20radiopharmaceutical%20extravasation,may%20go%20unnoticed%20until%20later>

<https://jnm.snmjournals.org/content/61/supplement_1/1018#:~:text=Conclusions%3A%20Diagnostic%20radiopharmaceutical%20extravasations%20can,radiopharmaceutical%20extravasations%20also%20cause%20harm>

<https://pubmed.ncbi.nlm.nih.gov/23835019/#:~:text=Radiopharmaceuticals%20extravasation%20is%20rare%20but,of%20extravasation%20of%20these%20drugs>

**3^rd^ ChatGPT response**

Radiopharmaceutical extravasation, which occurs when a radiopharmaceutical is inadvertently injected into the tissues surrounding a vein rather than directly into the vein itself, can have various effects on a patient. These effects depend on the type and amount of radiopharmaceutical involved. Here are some potential impacts:

1. **Localized Radiation Injury**: The most significant concern with extravasation is localized radiation injury to the tissue surrounding the injection site. This is because the radiopharmaceutical emits radiation, which can damage nearby tissue.
2. **Altered Imaging Results**: In diagnostic procedures, extravasation can lead to misleading or unclear imaging results. This is because the radiopharmaceutical is not properly distributed in the body, affecting the accuracy of the diagnostic images.
3. **Pain and Inflammation**: The patient may experience pain, redness, and swelling at the site of the extravasation. This is a reaction to both the chemical nature of the radiopharmaceutical and the physical injury from the injection.
4. **Delayed Healing**: The healing process of the affected tissue might be slower due to the localized radiation damage.
5. **Risk of Necrosis**: In severe cases, especially with high doses of radiopharmaceuticals, there's a risk of tissue necrosis (death of tissue) in the area surrounding the extravasation.
6. **Infection**: Any break in skin integrity, such as that caused by an extravasation, increases the risk of infection.
7. **Psychological Impact**: The experience of extravasation and any complications arising from it can cause anxiety and distress to the patient.

The management of radiopharmaceutical extravasation involves immediate care to reduce the spread of the substance, monitoring for adverse effects, and possibly administering treatments to mitigate tissue damage. It's essential for healthcare professionals to be vigilant about this potential complication and to provide appropriate patient care and follow-up.

References

<https://jnm.snmjournals.org/content/61/supplement_1/1018#:~:text=Conclusions%3A%20Diagnostic%20radiopharmaceutical%20extravasations%20can,radiopharmaceutical%20extravasations%20also%20cause%20harm>

<https://www.frontiersin.org/articles/10.3389/fmed.2021.684157/full#:~:text=Although%20an%20extravasation%20is%20typically,ionizing%20radiation%20on%20patient%20tissue>

<https://pubmed.ncbi.nlm.nih.gov/35085120/#:~:text=Immediate%20symptoms%20of%20radiopharmaceutical%20extravasation,may%20go%20unnoticed%20until%20later>

<https://journals.lww.com/health-physics/Fulltext/2022/11000/Practical_Tools_for_Patient_specific.1.aspx#:~:text=Tissue,radiopharmaceutical%20used%2C%20and%20residence%20time>

**Question 9.**

**Are radiopharmaceutical extravasations harmful?**

**1^st^ ChatGPT response**

Radiopharmaceutical extravasations, which occur when radiopharmaceuticals used in nuclear medicine accidentally infiltrate into the tissues surrounding a vein, are generally considered a minor complication but can have varying degrees of harm depending on several factors.

1. **Severity of Extravasation**: The extent of harm is influenced by the amount of radiopharmaceutical that has extravasated, the specific radiopharmaceutical used, and the rate of extravasation. In most cases, the extravasation is small and resolves without significant tissue damage.
2. **Type of Radiopharmaceutical**: Some radiopharmaceuticals are more likely to cause tissue damage than others. For example, radiopharmaceuticals used for therapy (like radioiodine for thyroid cancer) are more likely to cause harm compared to those used for diagnostic purposes.
3. **Patient Factors**: Individual patient factors like the health of the veins, the presence of underlying medical conditions, and the body's response to the extravasated material can also influence the extent of harm.
4. **Management and Intervention**: Prompt recognition and appropriate management of extravasation can minimize tissue damage. This might include elevating the affected limb, applying a cold or warm compress (depending on the type of radiopharmaceutical), and monitoring the site for any signs of inflammation or injury.

In summary, while radiopharmaceutical extravasations can potentially be harmful, the actual risk of significant harm is typically low, especially when managed properly. However, it is important to follow safety protocols to minimize the risk of extravasation and to manage it effectively if it occurs.

References

<https://www.ncbi.nlm.nih.gov/pmc/articles/PMC5434120/#:~:text=Extravasation%20of%20therapeutic%20radiopharmaceuticals%20can,immediate%20care%20of%20radiopharmaceutical%20extravasation>

<https://jnm.snmjournals.org/content/61/supplement_1/1018#:~:text=Conclusions%3A%20Diagnostic%20radiopharmaceutical%20extravasations%20can,dosimetric%20calculations%20of%20extravasated>

<https://pubmed.ncbi.nlm.nih.gov/23835019/#:~:text=Management%20should%20be%20immediate,indelible%20pen%2C%20informing%20the%20doctor>

**2^nd^ ChatGPT Response**

Radiopharmaceutical extravasations refer to the accidental leakage of radiopharmaceuticals (radioactive materials used in medical imaging and therapy) into the tissue surrounding a vein during injection. The extent of harm from a radiopharmaceutical extravasation can vary based on several factors:

1. **Type and Activity of the Radiopharmaceutical**: Some agents are more radiotoxic than others, and higher activities (amounts) of radiation can increase the potential for damage.
2. **Extravasation Volume**: Larger volumes can spread to more extensive areas of tissue, potentially causing more harm.
3. **Tissue Sensitivity**: Some tissues are more sensitive to radiation than others. For example, extravasation in areas with rapidly dividing cells or in close proximity to critical structures may be more problematic.
4. **Duration of Exposure**: Longer exposure times can lead to more significant tissue damage.

Typically, small extravasations might not cause significant harm and may resolve on their own, leading to minimal or no long-term effects. However, significant extravasations, especially of certain high-energy or high-dose radiopharmaceuticals, can cause tissue damage, leading to pain, swelling, and in severe cases, necrosis or functional impairment.

In clinical settings, protocols are in place to minimize the risk of extravasation and to manage it promptly if it occurs. Patients undergoing procedures involving radiopharmaceuticals are monitored for signs of extravasation, and medical staff are trained to respond appropriately.

If you have concerns about radiopharmaceutical extravasation, it's important to consult with medical professionals who can provide advice based on the specific circumstances and substances involved.

References

<https://www.ncbi.nlm.nih.gov/pmc/articles/PMC5434120/#:~:text=Extravasation%20of%20therapeutic%20radiopharmaceuticals%20can,immediate%20care%20of%20radiopharmaceutical%20extravasation>

<https://jnm.snmjournals.org/content/61/supplement_1/1018#:~:text=Conclusions%3A%20Diagnostic%20radiopharmaceutical%20extravasations%20can,dosimetric%20calculations%20of%20extravasated>

<https://www.frontiersin.org/articles/10.3389/fmed.2021.684157/full#:~:text=Conclusions%3A%20Our%20findings%20suggest%20that,limits%20and%20deterministic%20effect%20thresholds>

<https://ejnmmiphys.springeropen.com/articles/10.1186/s40658-021-00378-3#:~:text=Extravasation%20of%20radiopharmaceuticals%20used%20for,dose%20estimation%20in%20the%20literature>

**3^rd^ ChatGPT response**

Radiopharmaceutical extravasations, which occur when radiopharmaceuticals are inadvertently injected into the tissue surrounding a vein rather than directly into the vein itself, can potentially be harmful, depending on several factors:

1. **Type of Radiopharmaceutical**: Some radiopharmaceuticals are more irritative or radioactive than others. Those with higher radiation energy or those that are known irritants can cause more tissue damage.
2. **Amount of Extravasation**: A larger volume of extravasated material can increase the risk of local tissue damage.
3. **Location of Extravasation**: Certain areas of the body are more sensitive to radiation and can be more susceptible to damage.
4. **Patient's Overall Health**: Individuals with certain pre-existing conditions or compromised immune systems may be more vulnerable to complications.

While minor extravasations are usually not serious and resolve on their own, in some cases, they can lead to tissue damage, radiation burns, or necrosis. Proper injection techniques and immediate attention to any extravasation can minimize the risk of complications.

In clinical practice, if an extravasation is suspected, immediate measures are taken to reduce the local concentration of the radiopharmaceutical, such as elevating the affected limb and applying cold or warm compresses, depending on the type of radiopharmaceutical involved. It's also important to monitor the site for signs of inflammation or other adverse effects.

For a more detailed understanding of the risks and management strategies associated with radiopharmaceutical extravasations, consulting a nuclear medicine professional or referring to specific clinical guidelines is advisable.

References

<https://www.ncbi.nlm.nih.gov/pmc/articles/PMC5434120/#:~:text=Radiopharmaceutical%20extravasation%20can%20potentially%20lead,interventions%2C%20and%20effectiveness%20of%20these>

<https://pubmed.ncbi.nlm.nih.gov/28303300/#:~:text=Extravasation%20of%20therapeutic%20radiopharmaceuticals%20can,immediate%20care%20of%20radiopharmaceutical%20extravasation>

<https://www.ncbi.nlm.nih.gov/pmc/articles/PMC7431942/#:~:text=At%20the%20first%20sign%20of,1>

<https://journals.lww.com/health-physics/Abstract/2022/04000/Radiopharmaceutical_Extravasation__Pragmatic.15.aspx#:~:text=f%20misadministration%20occurs%2C%20called%20an,Pragmatic%20elements%20of>

<https://pubmed.ncbi.nlm.nih.gov/28303300/>

<https://www.frontiersin.org/articles/10.3389/fnume.2023.1219202/full>

<https://pubmed.ncbi.nlm.nih.gov/28303300/>

<https://www.frontiersin.org/articles/10.3389/fnume.2023.1219202/full>

**Question 10.**

**Are there any long-term effects of radiopharmaceutical extravasations?**

**1^st^ ChatGPT response**

Radiopharmaceutical extravasations, where a radioactive drug used for diagnostic or therapeutic purposes leaks into surrounding tissues during intravenous administration, can have potential long-term effects, although these are generally rare and depend on various factors. The extent of the effects largely depends on the type and amount of radiopharmaceutical involved, the rate of local dispersion and systemic absorption, and the radiosensitivity of the affected tissue.

1. **Local Tissue Damage**: The most common long-term effect is localized tissue damage, which can manifest as radiation burns, necrosis, or scarring. This damage is typically confined to the area of extravasation.
2. **Radiation Induced Neoplasms**: There is a theoretical risk of radiation-induced neoplasms in the area of extravasation, although this is considered extremely rare. This risk is influenced by the type of radiopharmaceutical and the dose received by the tissues.
3. **Radiation Fibrosis**: In some cases, radiation fibrosis can occur in the tissues surrounding the extravasation site. This can lead to stiffness and limited mobility, especially if the extravasation occurs near a joint.
4. **Delayed Wound Healing**: Radiation can impair the healing process, so if an extravasation occurs, it may take longer for the affected area to heal.
5. **Edema and Pain**: Persistent edema (swelling) and pain can occur at the extravasation site, which may last for an extended period.
6. **Secondary Effects**: Depending on the location and severity of the extravasation, secondary effects such as nerve damage or reduced blood flow to the affected area might occur.

It's important to note that immediate and appropriate intervention following an extravasation can significantly reduce the risk and severity of long-term effects. This includes measures like stopping the injection immediately, attempting to aspirate the extravasated fluid, elevating and immobilizing the affected limb, and applying cold or warm compresses depending on the type of radiopharmaceutical.

In clinical practice, radiopharmaceutical extravasations are rare and their long-term effects are not commonly observed due to the low volumes and activity typically used in diagnostic imaging. However, it's crucial for healthcare providers to be aware of these potential effects and to take steps to prevent extravasation and to manage it effectively if it occurs.

References

<https://pubmed.ncbi.nlm.nih.gov/28303300/>

<https://www.frontiersin.org/articles/10.3389/fnume.2023.1219202/full>

**2^nd^ ChatGPT Response**

Radiopharmaceutical extravasation, where a radioactive drug used for imaging or treatment leaks out of the blood vessel into the surrounding tissue, can have potential long-term effects, although they are generally rare. The severity and nature of these effects often depend on several factors such as the type of radiopharmaceutical used, the amount of leakage, the location of extravasation, and the patient's overall health.

1. **Tissue Damage:** One of the most common long-term effects is localized tissue damage. The radiation can cause injury to the skin and underlying tissues, potentially leading to necrosis or scarring.
2. **Radiation Burns:** Although rare, severe extravasations can lead to radiation burns which may take a long time to heal and could lead to long-term skin changes.
3. **Altered Drug Distribution:** Extravasation can affect the distribution of the drug within the body, potentially leading to suboptimal diagnostic or therapeutic results and the need for repeat procedures.
4. **Radiation-induced Neoplasms:** There is a very low risk, but it's theoretically possible, that exposure to radiation could lead to the development of neoplasms in the affected area many years later.
5. **Functional Impairment:** If extravasation occurs near a joint or a critical area, it might lead to long-term functional impairment due to tissue damage or scarring.

Immediate management of an extravasation involves stopping the injection, attempting to remove as much of the radiopharmaceutical as possible, and minimizing radiation exposure to the affected area. Long-term effects are more likely if the extravasation is not promptly and effectively managed. Regular monitoring and medical care are crucial in managing potential long-term effects. As with any medical procedure, it's important to weigh the benefits of radiopharmaceuticals against the potential risks. If you suspect or have experienced an extravasation, consult your healthcare provider immediately for appropriate care and monitoring.

References

<https://pubmed.ncbi.nlm.nih.gov/28303300/>

<https://jnm.snmjournals.org/content/61/supplement_1/1018>

**3^rd^ ChatGPT response**

Radiopharmaceutical extravasations, which refer to the leakage of radiopharmaceuticals into the surrounding tissue during intravenous administration, can have potential long-term effects, although these are generally rare and often depend on the specific type and amount of radiopharmaceutical involved. The long-term effects may include:

1. **Local Tissue Damage**: High radiation doses to local tissues can cause damage, potentially leading to necrosis (tissue death) or fibrosis (thickening and scarring of tissue).
2. **Radiation Burns**: In severe cases, radiation burns may occur, though this is more common with high-dose radiopharmaceuticals.
3. **Altered Lymphatic Drainage**: If the extravasation occurs near a lymph node, it may alter lymphatic drainage patterns. This is particularly relevant in lymphoscintigraphy, a procedure used to identify sentinel lymph nodes prior to surgery in cancers like breast cancer.
4. **Radiation-Induced Neoplasms**: There is a theoretical risk of radiation-induced neoplasms (tumors) due to local high radiation doses, but this is extremely rare.
5. **Functional Impairment**: Depending on the location of extravasation, there might be functional impairment of the affected limb or organ, especially if significant tissue damage occurs.

It's important to note that the likelihood and severity of long-term effects are influenced by factors such as the radiopharmaceutical's physical properties, the dose administered, and the effectiveness of immediate management after extravasation. Prompt recognition and management of an extravasation incident are crucial in minimizing potential long-term effects. Healthcare providers should always follow best practices for intravenous administration of radiopharmaceuticals to reduce the risk of extravasation.

References

<https://pubmed.ncbi.nlm.nih.gov/28303300/>

<https://jnm.snmjournals.org/content/61/supplement_1/1018>

<https://pubmed.ncbi.nlm.nih.gov/36265907/>

**Question 11.**

**Are there any systemic effects of radiopharmaceutical extravasations?**

**1^st^ ChatGPT response**

Radiopharmaceutical extravasation refers to the accidental leakage of a radiopharmaceutical agent (used in medical imaging or therapy) into the surrounding tissues during intravenous administration. The systemic effects of radiopharmaceutical extravasation depend on several factors, including the type of radiopharmaceutical used, the amount extravasated, and the site of extravasation. Here are some key points:

1. **Type of Radiopharmaceutical**: The systemic effects vary depending on the radiopharmaceutical involved. Some agents are more prone to cause local tissue reactions, while others might have systemic effects if absorbed in significant amounts.
2. **Amount Extravasated**: Small volumes of extravasation might not lead to significant systemic effects, while larger volumes can increase the risk of both local and systemic adverse effects.
3. **Local Effects**: The most common immediate concern with extravasation is local tissue reactions. These can include pain, swelling, redness, and tissue necrosis in severe cases. Some radiopharmaceuticals are more irritative to tissues and may cause more significant local reactions.
4. **Systemic Effects**: Systemic effects are less common but can occur, especially with larger extravasations or with certain agents. These might include allergic reactions, systemic toxicity affecting various organs, and altered biodistribution of the radiopharmaceutical, which can affect diagnostic accuracy or therapeutic efficacy.
5. **Delayed Effects**: In cases involving radioactive materials, there is also a concern about long-term radiation exposure to the local tissues, which could potentially increase the risk of radiation-induced damage or malignancy.
6. **Management and Monitoring**: Management of extravasation typically involves measures to minimize local tissue damage and monitoring for any systemic effects. This may include local cooling, elevation of the affected limb, and in some cases, administration of specific antidotes or treatments to mitigate the effects of the radiopharmaceutical.

In clinical practice, the risk of significant systemic effects from radiopharmaceutical extravasation is generally low, especially with adherence to proper injection techniques and immediate management of any extravasation incidents. However, each case should be evaluated individually, considering the specific radiopharmaceutical and the circumstances of the extravasation.

References

<https://pubmed.ncbi.nlm.nih.gov/28303300/>

<https://pubmed.ncbi.nlm.nih.gov/35085120/>

<https://pubmed.ncbi.nlm.nih.gov/23835019/>

**2^nd^ ChatGPT Response**

Radiopharmaceutical extravasation refers to the accidental leakage of a radiopharmaceutical — a radioactive compound used in medical imaging or treatment — into the surrounding tissue instead of the intended target, typically a vein. This can occur during procedures like positron emission tomography (PET) scans or therapeutic administrations. The systemic effects of such extravasations largely depend on several factors, including the type and activity of the radiopharmaceutical, the volume extravasated, and the patient's overall health. Here are some potential systemic effects and considerations:

1. **Local Tissue Effects**: The most immediate concern is usually the local tissue reaction. High radiation doses can cause radiation burns and tissue necrosis. However, systemic effects might not be prominent if the extravasation is small and contained.
2. **Radiation Dose Redistribution**: If a radiopharmaceutical designed to target a specific organ or tumor leaks into surrounding tissues, it may result in a lower than intended dose to the target area and an increased dose to the unintended area, potentially affecting the diagnostic accuracy or therapeutic efficacy.
3. **Whole-Body Exposure**: For certain high-energy radiopharmaceuticals, there can be a concern for whole-body exposure if the compound redistributes systemically. This could potentially lead to systemic effects, especially with repeated or high-dose exposures.
4. **Delayed Effects**: Long-term effects, such as the development of secondary malignancies or tissue damage, can be a concern, particularly with therapeutic radiopharmaceuticals that deliver high doses of radiation.
5. **Individual Variability**: Patients with certain pre-existing conditions may be more susceptible to the effects of radiation, and thus even minor extravasations could have more pronounced systemic effects.

In most cases, especially with diagnostic procedures, the doses are relatively low, and systemic effects are minimal. However, monitoring and immediate intervention are crucial to minimize local tissue damage and prevent potential systemic effects. If extravasation occurs, it's important to follow established protocols which might include local cooling, elevation of the affected limb, and possibly the administration of specific treatments to facilitate the redistribution or removal of the radiopharmaceutical.

References

<https://www.frontiersin.org/articles/10.3389/fnume.2023.1219202/full>

**3^rd^ ChatGPT response**

Radiopharmaceutical extravasation refers to the leakage of radiopharmaceuticals into the surrounding tissues during intravenous administration. These incidents are relatively rare but can occur during nuclear medicine procedures. The systemic effects of radiopharmaceutical extravasation depend on various factors, including the type and quantity of the radiopharmaceutical, the site of extravasation, and the patient's overall health. Generally, the systemic effects are minimal due to the small quantities used in diagnostic procedures. However, potential concerns include:

1. **Localized Radiation Exposure:** The primary effect is usually localized to the area of extravasation. This can lead to higher radiation doses in the affected tissue, potentially causing local tissue damage.
2. **Altered Diagnostic Accuracy:** In diagnostic procedures, extravasation can lead to inaccurate imaging results, as the radiopharmaceutical may not reach its intended target in the body.
3. **Delayed Absorption:** Some of the extravasated radiopharmaceutical might slowly be absorbed into the systemic circulation, potentially causing delayed or altered distribution, which can affect diagnostic imaging or therapeutic efficacy.
4. **Therapeutic Radiopharmaceuticals:** For therapeutic radiopharmaceuticals, which are administered in higher doses compared to diagnostic ones, the risk of localized tissue damage is higher, and there could be a reduced therapeutic effect due to the loss of the agent at the intended target site.
5. **Long-term Effects:** Long-term effects are not well-characterized but could potentially include localized tissue fibrosis or necrosis, especially with higher doses of radiation.
6. **Infection and Inflammation:** There is also a risk of infection and inflammation at the site of extravasation, though this is more related to the procedure itself rather than the radiopharmaceutical.

Overall, while systemic effects are rare and usually not severe with diagnostic radiopharmaceuticals, careful injection techniques and monitoring are essential to minimize the risk of extravasation and its potential consequences. In cases of extravasation, prompt intervention to reduce localized radiation dose is recommended.

References

<https://pubmed.ncbi.nlm.nih.gov/28303300/>

<https://jnm.snmjournals.org/content/61/supplement_1/1018>

**Question 12.**

**Should a patient be told when they have been extravasated with a radiopharmaceutical?**

**1^st^ ChatGPT response**

When a patient experiences extravasation (the leakage of a fluid, in this case, a radiopharmaceutical, into the surrounding tissue) during a medical procedure, it is generally considered best practice to inform the patient about the incident. There are several reasons for this:

1. **Ethical Considerations**: Honesty in healthcare is a fundamental ethical principle. Patients have the right to know about all aspects of their care, including any complications or errors.
2. **Medical Management**: If extravasation occurs, the patient may need to be monitored for any adverse reactions or complications. Informing the patient allows them to be aware of potential symptoms and seek medical attention if necessary.
3. **Patient Autonomy**: Patients have the right to make informed decisions about their healthcare. Knowing about the extravasation can impact their choices regarding future treatments or procedures.
4. **Legal and Regulatory Requirements**: In many jurisdictions, healthcare providers are legally required to inform patients about any significant medical events or errors that occur during their treatment.
5. **Building Trust**: Transparency in healthcare helps build trust between healthcare providers and patients. Concealing information about extravasation could damage this trust if the patient later finds out.

However, the way this information is conveyed should be handled sensitively and professionally, ensuring the patient understands the situation, the potential implications, and the steps being taken to manage it. Healthcare providers should also be prepared to answer any questions the patient may have and provide support as needed.

References

*** Did not provide references, just this background.

The response regarding the importance of informing a patient about extravasation with a radiopharmaceutical is based on general medical and ethical principles, rather than specific references. However, the key concepts can be supported by a variety of sources in medical ethics, patient care standards, and legal guidelines in healthcare. Here are the key points with the types of references that typically support them:

1. **Ethical Considerations**: Medical ethics literature often emphasizes the importance of patient autonomy and the right to be informed. Books and articles on medical ethics would provide insight into this.

2. **Informed Consent**: This is a fundamental concept in healthcare, covered extensively in medical legal textbooks and healthcare policy documents.

3. **Risk Communication**: The importance of communicating risks, including complications like extravasation, is covered in patient safety and communication guidelines, which can be found in healthcare quality improvement literature.

4. **Patient Care and Follow-up**: Clinical guidelines for managing extravasation, particularly with radiopharmaceuticals, would detail the standard of care and follow-up procedures. These are often found in medical journals or institutional protocols.

5. **Medical and Legal Implications**: Legal textbooks and case law reviews regarding patient rights and medical liability would provide information on the legal aspects of patient communication after adverse events.

6. **Documentation**: This aspect is a standard practice in healthcare, often covered in clinical practice guidelines and medical record-keeping policies.

7. **Team Communication**: The importance of interdisciplinary communication in patient care is a topic covered in medical education and team management literature.

For the most accurate and detailed information, consulting specific medical journals, textbooks, and legal case studies related to radiopharmaceuticals, nuclear medicine, and healthcare law is recommended. Additionally, guidelines from relevant professional bodies and healthcare institutions would provide specific protocols and policies.

**2^nd^ ChatGPT Response**

Yes, a patient should be informed if they have experienced extravasation with a radiopharmaceutical. Extravasation is when a drug intended for the bloodstream leaks into the surrounding tissue. In the context of radiopharmaceuticals, which are radioactive compounds used primarily for diagnosis or treatment in nuclear medicine, extravasation can lead to localized radiation injury. Here’s why informing the patient is important:

1. **Ethical Consideration**: Patients have the right to be informed about their medical care. Knowing about an extravasation can help them understand any symptoms or issues they might experience.
2. **Symptom Monitoring**: Patients informed of the extravasation can be more vigilant about noticing and reporting any symptoms or changes in the affected area. This could include pain, swelling, redness, or any other unusual changes.
3. **Follow-up Care**: Knowledge of the extravasation might necessitate additional follow-up appointments, monitoring, or treatments to manage any potential complications. Early intervention can be crucial in preventing more severe damage.
4. **Informed Consent**: Understanding the risks, including the possibility and consequences of extravasation, is a part of the informed consent process for any procedure involving radiopharmaceuticals.
5. **Trust and Transparency**: Open communication about any complications can help maintain trust between the healthcare provider and the patient. It also contributes to the patient's understanding of their treatment and health.

In any healthcare setting, clear communication and patient safety are paramount. While not all extravasations lead to serious complications, the potential for localized radiation injury with radiopharmaceuticals makes it especially important to inform and appropriately manage any cases that do occur.

References

*** Did not provide references, just this background.

The response regarding the importance of informing a patient about extravasation with a radiopharmaceutical is based on general medical and ethical principles, rather than specific references. However, the key concepts can be supported by a variety of sources in medical ethics, patient care standards, and legal guidelines in healthcare. Here are the key points with the types of references that typically support them:

1. **Ethical Considerations**: Medical ethics literature often emphasizes the importance of patient autonomy and the right to be informed. Books and articles on medical ethics would provide insight into this.

2. **Informed Consent**: This is a fundamental concept in healthcare, covered extensively in medical legal textbooks and healthcare policy documents.

3. **Risk Communication**: The importance of communicating risks, including complications like extravasation, is covered in patient safety and communication guidelines, which can be found in healthcare quality improvement literature.

4. **Patient Care and Follow-up**: Clinical guidelines for managing extravasation, particularly with radiopharmaceuticals, would detail the standard of care and follow-up procedures. These are often found in medical journals or institutional protocols.

5. **Medical and Legal Implications**: Legal textbooks and case law reviews regarding patient rights and medical liability would provide information on the legal aspects of patient communication after adverse events.

6. **Documentation**: This aspect is a standard practice in healthcare, often covered in clinical practice guidelines and medical record-keeping policies.

7. **Team Communication**: The importance of interdisciplinary communication in patient care is a topic covered in medical education and team management literature.

For the most accurate and detailed information, consulting specific medical journals, textbooks, and legal case studies related to radiopharmaceuticals, nuclear medicine, and healthcare law is recommended. Additionally, guidelines from relevant professional bodies and healthcare institutions would provide specific protocols and policies.

**3^rd^ ChatGPT response**

In medical practice, patient communication and informed consent are critical aspects of care, especially when dealing with complications or adverse events such as extravasation of a radiopharmaceutical. Extravasation refers to the leakage of a drug or substance from a vein into the surrounding tissue during intravenous administration. When it comes to radiopharmaceuticals, which are radioactive compounds used in nuclear medicine, extravasation can potentially lead to local radiation injury.

The general principles for handling such situations include:

1. **Ethical Considerations**: Ethically, patients have the right to know about their medical conditions and any complications arising from treatments. This is rooted in the principle of autonomy, allowing patients to make informed decisions about their care.

2. **Informed Consent**: Informing a patient about extravasation falls under the broader umbrella of informed consent, where patients are made aware of potential risks and outcomes of medical procedures.

3. **Risk Communication**: If extravasation occurs, the healthcare provider should inform the patient about the incident, including potential risks, the severity of the extravasation, and any steps taken to mitigate harm.

4. **Patient Care and Follow-up**: Effective communication also involves discussing the care plan and follow-up measures to address the extravasation. This may include monitoring, treatment of any local reaction, and measures to prevent complications.

5. **Medical and Legal Implications**: From a legal standpoint, failure to inform a patient about such an incident could have implications in terms of medical liability. Transparent communication is essential for maintaining trust and avoiding legal issues.

6. **Documentation**: It's crucial to document the incident, patient communication, and any measures taken in response to the extravasation.

7. **Team Communication**: Ensuring that all members of the healthcare team are aware of the extravasation can aid in consistent and effective patient care.

It's important to note that specific protocols may vary depending on the healthcare institution, the type of radiopharmaceutical involved, and local regulations. Consulting institutional policies and guidelines is always recommended. Additionally, involving a multidisciplinary team, including radiologists, nuclear medicine specialists, and pharmacists, can help in managing such situations effectively.

References

*** Did not provide references, just this background.

The response regarding the importance of informing a patient about extravasation with a radiopharmaceutical is based on general medical and ethical principles, rather than specific references. However, the key concepts can be supported by a variety of sources in medical ethics, patient care standards, and legal guidelines in healthcare. Here are the key points with the types of references that typically support them:

1. **Ethical Considerations**: Medical ethics literature often emphasizes the importance of patient autonomy and the right to be informed. Books and articles on medical ethics would provide insight into this.

2. **Informed Consent**: This is a fundamental concept in healthcare, covered extensively in medical legal textbooks and healthcare policy documents.

3. **Risk Communication**: The importance of communicating risks, including complications like extravasation, is covered in patient safety and communication guidelines, which can be found in healthcare quality improvement literature.

4. **Patient Care and Follow-up**: Clinical guidelines for managing extravasation, particularly with radiopharmaceuticals, would detail the standard of care and follow-up procedures. These are often found in medical journals or institutional protocols.

5. **Medical and Legal Implications**: Legal textbooks and case law reviews regarding patient rights and medical liability would provide information on the legal aspects of patient communication after adverse events.

6. **Documentation**: This aspect is a standard practice in healthcare, often covered in clinical practice guidelines and medical record-keeping policies.

7. **Team Communication**: The importance of interdisciplinary communication in patient care is a topic covered in medical education and team management literature.

For the most accurate and detailed information, consulting specific medical journals, textbooks, and legal case studies related to radiopharmaceuticals, nuclear medicine, and healthcare law is recommended. Additionally, guidelines from relevant professional bodies and healthcare institutions would provide specific protocols and policies.

**Question 13.**

**Can a radiopharmaceutical extravasation affect a patient’s diagnostic image?**

**1^st^ ChatGPT response**

Yes, radiopharmaceutical extravasation can affect a patient's diagnostic image. Extravasation refers to the leakage of a radiopharmaceutical agent from the intended intravenous (IV) site into the surrounding tissue. This can happen during the administration of a radiotracer for diagnostic imaging procedures such as Positron Emission Tomography (PET) or Single Photon Emission Computed Tomography (SPECT).

The impact of extravasation on the diagnostic image depends on several factors:

1. **Type of Radiopharmaceutical**: Different agents have varying tissue binding characteristics. Some may stay localized at the extravasation site, while others may redistribute.
2. **Extent of Extravasation**: A small amount of leakage may have a minimal impact, but a significant extravasation can cause a local increase in radioactivity which may lead to artifacts or false-positive findings on the scan.
3. **Location of Extravasation**: If extravasation occurs near the area of interest, it can interfere with the interpretation of the scan. For example, extravasation in the arm during a cardiac PET scan might be misinterpreted as abnormal cardiac activity.
4. **Time of Detection**: If extravasation is detected early, measures can be taken to minimize its impact, such as applying a warm or cold compress to disperse or immobilize the radiopharmaceutical.

Overall, while radiopharmaceutical extravasation may not always significantly impact the diagnostic quality of an image, it is a complication that needs to be addressed promptly to ensure accurate interpretation of imaging studies.

References

<https://jnm.snmjournals.org/content/61/supplement_1/1018>

<https://www.frontiersin.org/articles/10.3389/fnume.2023.1171918/full>

<https://pubmed.ncbi.nlm.nih.gov/28303300/>

**2^nd^ ChatGPT response**

Yes, radiopharmaceutical extravasation can affect a patient's diagnostic image significantly. Extravasation is the leakage of a radiopharmaceutical into the surrounding tissue instead of the intended circulatory system or target tissue. This can lead to several issues:

1. **Reduced Diagnostic Quality**: The leaked radiopharmaceutical might not reach the target organ in the expected concentrations, leading to suboptimal or misleading imaging results. This can make it difficult to accurately diagnose or assess the extent of a disease.
2. **False Positives/Negatives**: Accumulation of the radiopharmaceutical in non-target tissues may appear as unexpected hot spots on the scan, potentially leading to false-positive results. Conversely, the reduced delivery to the intended site might result in a false-negative outcome.
3. **Dosimetry Concerns**: Extravasation can lead to a higher localized radiation dose to the surrounding tissue, which might not be accounted for in the standard dosimetry calculations. This can raise concerns about radiation-induced tissue damage.
4. **Delayed or Repeated Procedures**: If an extravasation significantly compromises the quality of the diagnostic image, the scan might need to be delayed or repeated, leading to increased radiation exposure, inconvenience, and potentially higher costs for the patient.

In practice, if extravasation is suspected or observed, the medical team might take immediate steps to mitigate its effects, such as applying gentle pressure to the area or using a cold or warm compress, depending on the type of radiopharmaceutical used. The impact of extravasation also depends on the volume and type of radiopharmaceutical involved, the location of the extravasation, and the patient's individual characteristics. Monitoring and appropriate management are essential to minimize any potential negative effects on diagnostic imaging and patient safety.

References

<https://jnm.snmjournals.org/content/61/supplement_1/1018>

<https://www.frontiersin.org/articles/10.3389/fnume.2023.1171918/full>

**3^rd^ ChatGPT response**

Yes, radiopharmaceutical extravasation can affect a patient's diagnostic image. Radiopharmaceuticals are special drugs used in nuclear medicine imaging, like PET or SPECT scans, and they work by emitting radiation that is detected by imaging devices to create detailed images of the body's internal structures.

When a radiopharmaceutical is administered, it's usually injected into a vein. Extravasation occurs when this radiopharmaceutical leaks out of the vein into the surrounding tissue. This can lead to a concentration of the radiopharmaceutical in the extravasation area, causing a localized area of increased radiotracer uptake. This localized accumulation can create artifacts or false positives in the imaging, as it may appear as an abnormal area of increased activity that can be mistaken for a pathological finding.

Moreover, extravasation can result in reduced delivery of the radiopharmaceutical to the target tissue or organ, potentially leading to suboptimal or inconclusive imaging results. For example, in a cardiac perfusion scan, extravasation might lead to insufficient tracer in the heart, making it difficult to assess cardiac function accurately.

Healthcare professionals take various precautions to minimize the risk and impact of extravasation, including careful injection techniques and monitoring the injection site. If extravasation is suspected or detected, they may take additional steps, such as applying a cold or warm compress (depending on the radiopharmaceutical used) and elevating the affected limb, to help disperse the radiotracer more evenly.

References

<https://jnm.snmjournals.org/content/61/supplement_1/1018>

<https://pubmed.ncbi.nlm.nih.gov/28303300/>

**Question14.**

**How does an extravasation affect a patient’s therapeutic radiopharmaceutical procedure?**

**1^st^ ChatGPT response**

Extravasation during a therapeutic radiopharmaceutical procedure can significantly impact the patient's treatment, safety, and overall outcome. Radiopharmaceuticals are radioactive compounds used for treatment (therapeutic) or diagnostic purposes in nuclear medicine. These compounds are typically administered intravenously. Extravasation refers to the accidental leakage of these radiopharmaceuticals from the vein into the surrounding tissues during injection.

The impact of extravasation on a patient undergoing a therapeutic radiopharmaceutical procedure can vary depending on several factors, including the type and activity of the radiopharmaceutical, the volume extravasated, and the site of extravasation. Here are some of the potential effects and considerations:

1. **Localized Tissue Damage**: Radiopharmaceuticals are designed to deliver radiation to specific targets within the body. If a radiopharmaceutical extravasates, it can cause radiation damage to the non-targeted surrounding tissues, leading to inflammation, necrosis, or long-term tissue damage. The severity of the damage depends on the radiation dose and the radiopharmaceutical's properties.
2. **Reduced Therapeutic Efficacy**: Extravasation can result in a lower dose of the radiopharmaceutical reaching the intended target site, potentially reducing the effectiveness of the treatment. For therapies aimed at treating cancer or other diseases, this can impact the overall treatment outcome.
3. **Altered Biodistribution**: The leakage of the radiopharmaceutical into the surrounding tissue can alter its biodistribution, affecting both the therapeutic efficacy and the accuracy of any subsequent imaging done for assessment or planning purposes.
4. **Management and Treatment of Extravasation**: Immediate action is required to manage and mitigate the effects of extravasation. This may include stopping the injection, attempting to aspirate the extravasated fluid, applying cold or warm compresses (depending on the radiopharmaceutical used), and monitoring the affected area for any adverse effects. In some cases, specific interventions may be required to reduce the radiation dose to the affected tissue.
5. **Prevention and Training**: Preventing extravasation involves proper patient assessment, choosing the best venous access site, using appropriate injection techniques, and thorough training of the personnel administering the radiopharmaceuticals. Patient education on reporting any discomfort during the injection can also help in early detection.
6. **Follow-up and Monitoring**: Patients who experience extravasation may require close follow-up to monitor the affected area for signs of radiation-induced injury or tissue damage. This monitoring can help in timely intervention if complications develop.

In summary, extravasation during a therapeutic radiopharmaceutical procedure can have significant implications, affecting both the efficacy of the treatment and the safety of the patient. It necessitates immediate and appropriate management to minimize potential harm and ensure the best possible outcome for the patient.

References

No references were provided- When ChatGPT was asked to provide references, ChatGPT responded as the following:

The information provided in the response about the effects of extravasation during therapeutic radiopharmaceutical procedures and its management is based on general principles of nuclear medicine and radiopharmacy practice, as well as clinical safety guidelines. While I cannot cite specific documents because my training data includes a wide range of sources up to April 2023, including textbooks, clinical guidelines, and expert reviews in the field of nuclear medicine, radiology, and oncology, I can suggest types of references that typically contain such information:

1. **Clinical Guidelines and Consensus Statements**: Professional societies in nuclear medicine and radiology, such as the Society of Nuclear Medicine and Molecular Imaging (SNMMI), the European Association of Nuclear Medicine (EANM), and the American College of Radiology (ACR), often publish guidelines and consensus statements on the safe administration of radiopharmaceuticals and management of extravasation.

2. **Textbooks and Review Articles**: Textbooks on nuclear medicine and radiopharmacy often cover the principles of radiopharmaceutical administration, including the prevention and management of extravasation. Review articles in medical journals also discuss current practices and recommendations.

3. **Safety Manuals and Protocols**: Hospitals and nuclear medicine departments typically have safety manuals and protocols that include procedures for preventing and managing extravasation of radiopharmaceuticals.

For specific and up-to-date information, including guidelines on handling extravasation of particular radiopharmaceuticals, it is advisable to consult the latest publications from these sources or the product literature provided by the radiopharmaceutical manufacturers. Additionally, clinical practice resources available through academic journals and the websites of professional societies can provide further insights and detailed recommendations.

**2^nd^ ChatGPT Response**

Extravasation during a therapeutic radiopharmaceutical procedure can significantly affect the outcome and safety of the treatment. Radiopharmaceuticals are radioactive compounds used for therapy (treatment of diseases such as cancer) and diagnosis (imaging to detect diseases). When administered properly, these agents target specific tissues or organs, delivering radiation to diseased cells while sparing healthy tissues. Extravasation refers to the accidental leakage of the radiopharmaceutical from the intended vascular pathway (vein) into the surrounding tissue during injection or infusion.

The impact of extravasation in therapeutic radiopharmaceutical procedures includes:

1. **Reduced Efficacy of Treatment**: The primary concern is that the intended dose of radiation does not reach the target tissue or tumor. This can lead to a reduced therapeutic effect, potentially making the treatment less effective in managing the disease.
2. **Radiation Damage to Healthy Tissue**: Extravasation may result in a high concentration of radioactivity in the non-targeted area, causing radiation damage to healthy tissues. This can lead to local tissue necrosis (death of tissue), inflammation, and long-term damage, depending on the radiopharmaceutical's properties, the activity (dose), and the volume extravasated.
3. **Delayed Detection and Management**: Unlike extravasation with chemotherapeutic agents, which often causes immediate pain or skin changes, radiopharmaceutical extravasation might not be immediately noticeable. This can delay the detection and implementation of mitigation measures, increasing the risk of tissue damage.
4. **Management Challenges**: Once extravasation is detected, managing the effects can be challenging. Measures may include local cooling, elevation of the affected limb, and, in some cases, surgical intervention. However, the effectiveness of these interventions varies, and there is no universally accepted treatment protocol.
5. **Monitoring and Follow-Up**: Patients who experience extravasation may require additional monitoring and follow-up to assess and manage potential tissue damage. This can include visual assessments, symptom monitoring, and, in some cases, imaging studies to evaluate the extent of radiation exposure to non-target tissues.
6. **Psychological Impact**: Experiencing an extravasation event can be distressing for patients, leading to anxiety about the efficacy of their treatment and concerns about potential long-term effects.

Prevention of extravasation is a key aspect of patient care during the administration of radiopharmaceuticals. This includes careful selection of the injection site, use of appropriate injection techniques, and monitoring during and after the administration of the radiopharmaceutical. In cases where extravasation occurs, prompt identification and intervention are crucial to minimize potential harm.

References

No references were provided- When ChatGPT was asked to provide references, ChatGPT responded as the following:

The information provided in the response about the effects of extravasation during therapeutic radiopharmaceutical procedures and its management is based on general principles of nuclear medicine and radiopharmacy practice, as well as clinical safety guidelines. While I cannot cite specific documents because my training data includes a wide range of sources up to April 2023, including textbooks, clinical guidelines, and expert reviews in the field of nuclear medicine, radiology, and oncology, I can suggest types of references that typically contain such information:

1. **Clinical Guidelines and Consensus Statements**: Professional societies in nuclear medicine and radiology, such as the Society of Nuclear Medicine and Molecular Imaging (SNMMI), the European Association of Nuclear Medicine (EANM), and the American College of Radiology (ACR), often publish guidelines and consensus statements on the safe administration of radiopharmaceuticals and management of extravasation.

2. **Textbooks and Review Articles**: Textbooks on nuclear medicine and radiopharmacy often cover the principles of radiopharmaceutical administration, including the prevention and management of extravasation. Review articles in medical journals also discuss current practices and recommendations.

3. **Safety Manuals and Protocols**: Hospitals and nuclear medicine departments typically have safety manuals and protocols that include procedures for preventing and managing extravasation of radiopharmaceuticals.

For specific and up-to-date information, including guidelines on handling extravasation of particular radiopharmaceuticals, it is advisable to consult the latest publications from these sources or the product literature provided by the radiopharmaceutical manufacturers. Additionally, clinical practice resources available through academic journals and the websites of professional societies can provide further insights and detailed recommendations.

**3^rd^ ChatGPT response**

Extravasation during a therapeutic radiopharmaceutical procedure can significantly affect a patient's treatment and safety. Radiopharmaceuticals are radioactive compounds used for therapy in various diseases, including cancer. These compounds are designed to target specific tissues or cells within the body to deliver therapeutic radiation directly to the disease site, minimizing damage to surrounding healthy tissues. However, when extravasation occurs — the accidental leakage of the radiopharmaceutical from the blood vessel into the surrounding tissues during injection — it can lead to several issues:

1. **Reduced Efficacy**: The therapeutic effect of the radiopharmaceutical might be diminished because a portion of the intended dose does not reach the target tissue or organ. This reduction in delivered dose can lead to suboptimal treatment outcomes.
2. **Radiation Injury**: Extravasated radiopharmaceuticals can cause radiation damage to the surrounding tissues where they accumulate. The severity of tissue damage depends on the radiopharmaceutical's properties, the amount of extravasated material, and its half-life. Symptoms of radiation injury might include redness, swelling, blistering, or necrosis in severe cases.
3. **Delayed Diagnosis**: In diagnostic procedures, extravasation can lead to misinterpretation of the imaging results. Although this point is more relevant to diagnostic radiopharmaceuticals, it's worth noting that any procedure involving the injection of radioactive substances can be affected by extravasation.
4. **Management Challenges**: Managing extravasation incidents requires immediate attention. Measures might include stopping the injection, attempting to aspirate the extravasated fluid, applying cold or warm compresses (depending on the radiopharmaceutical used), and monitoring the affected area for signs of injury. In some cases, surgical intervention may be necessary to remove necrotic tissue.
5. **Increased Healthcare Costs**: The need for additional treatments to manage extravasation effects, potential surgical interventions, and extended hospital stays can lead to increased healthcare costs.

Prevention strategies are key to minimizing the risk of extravasation, including proper patient assessment, the use of experienced personnel for injection, careful selection of injection sites, and monitoring during and after the procedure. When extravasation occurs, prompt and appropriate management is critical to minimize tissue damage and manage patient discomfort.

References

No references were provided- When ChatGPT was asked to provide references, ChatGPT responded as the following:

The information provided in the response about the effects of extravasation during therapeutic radiopharmaceutical procedures and its management is based on general principles of nuclear medicine and radiopharmacy practice, as well as clinical safety guidelines. While I cannot cite specific documents because my training data includes a wide range of sources up to April 2023, including textbooks, clinical guidelines, and expert reviews in the field of nuclear medicine, radiology, and oncology, I can suggest types of references that typically contain such information:

1. **Clinical Guidelines and Consensus Statements**: Professional societies in nuclear medicine and radiology, such as the Society of Nuclear Medicine and Molecular Imaging (SNMMI), the European Association of Nuclear Medicine (EANM), and the American College of Radiology (ACR), often publish guidelines and consensus statements on the safe administration of radiopharmaceuticals and management of extravasation.

2. **Textbooks and Review Articles**: Textbooks on nuclear medicine and radiopharmacy often cover the principles of radiopharmaceutical administration, including the prevention and management of extravasation. Review articles in medical journals also discuss current practices and recommendations.

3. **Safety Manuals and Protocols**: Hospitals and nuclear medicine departments typically have safety manuals and protocols that include procedures for preventing and managing extravasation of radiopharmaceuticals.

For specific and up-to-date information, including guidelines on handling extravasation of particular radiopharmaceuticals, it is advisable to consult the latest publications from these sources or the product literature provided by the radiopharmaceutical manufacturers. Additionally, clinical practice resources available through academic journals and the websites of professional societies can provide further insights and detailed recommendations.

**Question 15.**

**Could a radiopharmaceutical extravasation alter the qualitative and quantitative results of a nuclear medicine procedure in a way that causes the patient to receive inappropriate or less than optimal care?**

**1^st^ ChatGPT response**

Yes, a radiopharmaceutical extravasation can potentially alter both the qualitative and quantitative results of a nuclear medicine procedure, leading to inappropriate or less than optimal care for the patient. Radiopharmaceutical extravasation refers to the leakage of a radiopharmaceutical agent from a blood vessel into the surrounding tissues during administration. This event can impact the distribution and localization of the radiotracer, affecting the imaging results in several ways:

1. **Quantitative Analysis:** For procedures that rely on quantitative measurements, such as dosimetry calculations for radionuclide therapy, extravasation can lead to inaccurate estimations of organ doses or treatment efficacy. This miscalculation may result in underdosing or overdosing of the radiopharmaceutical, potentially leading to suboptimal therapeutic outcomes or increased risk of side effects.
2. **Qualitative Analysis:** Extravasation can cause abnormal localization of the radiotracer outside of the target area, potentially mimicking pathological uptake or obscuring true pathological signals. In diagnostic imaging, such as PET or SPECT scans, this can lead to misinterpretation of the images, resulting in incorrect diagnoses or assessments of disease extent.
3. **Image Artifacts:** The localized concentration of radiotracer in the extravasation site can create image artifacts that interfere with the interpretation of nuclear medicine scans. These artifacts can obscure or simulate disease, complicating the diagnostic process.
4. **Patient Management:** Decisions based on inaccurately interpreted scans can lead to inappropriate patient management, including unnecessary additional testing, delayed treatment, or the selection of less effective therapeutic options.

Mitigation strategies include careful injection techniques, monitoring for signs of extravasation, and, if extravasation is detected, applying interventions such as gentle massage of the area (depending on the radiopharmaceutical and institution protocols) to disperse the agent more widely in the tissue. In some cases, recalculating doses or repeating the imaging procedure may be necessary to ensure accurate diagnosis and optimal patient care.

For accurate assessment and management, it's essential for nuclear medicine professionals to be aware of the potential impact of radiopharmaceutical extravasation and to incorporate strategies for its prevention, detection, and management into their practice.

References

No references were provided- When ChatGPT was asked to provide references, ChatGPT responded as the following:

My response is based on general principles and knowledge within the field of nuclear medicine, as my last training data update was in April 2023, and I don't have the ability to access or cite specific, up-to-date references directly. However, I can guide you on where to find authoritative sources for further reading:

1. **Textbooks on Nuclear Medicine**: Look for comprehensive textbooks that cover the principles of nuclear medicine, radiopharmacy, and the technical aspects of nuclear imaging. These texts often discuss the handling of radiopharmaceuticals, including the prevention and management of extravasation.

2. **Professional Guidelines and Position Statements**: Organizations such as the Society of Nuclear Medicine and Molecular Imaging (SNMMI) in the United States, the European Association of Nuclear Medicine (EANM), and others publish guidelines and position statements on best practices in nuclear medicine. These documents may include sections on radiopharmaceutical injection techniques, extravasation prevention, and management protocols.

3. **Scientific Journals**: Peer-reviewed journals focused on nuclear medicine, radiology, and medical imaging frequently publish research articles, reviews, and case reports on the implications of radiopharmaceutical extravasation. Examples include "The Journal of Nuclear Medicine," "European Journal of Nuclear Medicine and Molecular Imaging," and "Clinical Nuclear Medicine."

4. **Online Medical and Scientific Databases**: Databases such as PubMed, Scopus, or Web of Science allow you to search for articles related to radiopharmaceutical extravasation and its impact on imaging and therapy in nuclear medicine. Using search terms like "radiopharmaceutical extravasation," "nuclear medicine," and "imaging artifacts" can help you find relevant studies and reviews.

For the most accurate and up-to-date information, consulting these types of sources will be beneficial. Additionally, guidelines and protocols from specific healthcare institutions may also provide insights into how extravasation is managed in clinical practice.

**2^nd^ ChatGPT Response**

Yes, radiopharmaceutical extravasation can significantly affect the qualitative and quantitative results of a nuclear medicine procedure, potentially leading to inappropriate or less than optimal care for the patient. Here's how:

Qualitative Impact:

1. **Image Artifacts:** Extravasation of radiopharmaceuticals at the injection site can cause increased local radiation concentration, leading to artifacts on the imaging study. These artifacts can obscure or mimic pathology, complicating interpretation.
2. **Misinterpretation:** Especially in procedures like sentinel lymph node mapping, extravasation can lead to false-negative or false-positive findings. For example, if the radiopharmaceutical intended for lymphatic mapping remains at the injection site, it may not accurately reflect the lymphatic drainage pattern, potentially missing metastatic nodes.

Quantitative Impact:

1. **Dosimetry Calculations:** For therapies involving radiopharmaceuticals, such as radionuclide therapy for thyroid cancer or neuroendocrine tumors, accurate dosimetry calculations are crucial for effective treatment. Extravasation can lead to incorrect estimations of the dose delivered to the target tissue, resulting in suboptimal therapy or increased risk of toxicity to non-target tissues.
2. **Reduced Diagnostic Accuracy:** In quantitative studies, such as myocardial perfusion imaging or PET quantification, extravasation can result in underestimation of tracer uptake by the target organ. This could lead to underestimation of disease extent or function, affecting clinical decision-making.

Clinical Implications:

- **Delayed Diagnosis or Misdiagnosis:** Artifacts or misinterpretations caused by extravasation can lead to delayed diagnosis or misdiagnosis, affecting patient management and outcomes.
- **Treatment Efficacy:** For therapeutic procedures, inaccurate dose delivery to the target tissue can compromise treatment efficacy, potentially necessitating additional interventions or leading to progression of the disease.
- **Increased Patient Risk:** Incorrect dosimetry can also increase the risk of adverse effects from radiation exposure to non-target tissues.

Management Strategies:

To mitigate these impacts, it's essential to employ strategies like careful injection technique, monitoring for extravasation, and, if extravasation is detected, considering alternative imaging strategies or dosimetry adjustments. In some cases, repeating the imaging study or using a different imaging modality might be necessary to ensure accurate diagnosis and optimal patient care.

References

No references were provided- When ChatGPT was asked to provide references, ChatGPT responded as the following:

My response is based on general principles and knowledge within the field of nuclear medicine, as my last training data update was in April 2023, and I don't have the ability to access or cite specific, up-to-date references directly. However, I can guide you on where to find authoritative sources for further reading:

1. **Textbooks on Nuclear Medicine**: Look for comprehensive textbooks that cover the principles of nuclear medicine, radiopharmacy, and the technical aspects of nuclear imaging. These texts often discuss the handling of radiopharmaceuticals, including the prevention and management of extravasation.

2. **Professional Guidelines and Position Statements**: Organizations such as the Society of Nuclear Medicine and Molecular Imaging (SNMMI) in the United States, the European Association of Nuclear Medicine (EANM), and others publish guidelines and position statements on best practices in nuclear medicine. These documents may include sections on radiopharmaceutical injection techniques, extravasation prevention, and management protocols.

3. **Scientific Journals**: Peer-reviewed journals focused on nuclear medicine, radiology, and medical imaging frequently publish research articles, reviews, and case reports on the implications of radiopharmaceutical extravasation. Examples include "The Journal of Nuclear Medicine," "European Journal of Nuclear Medicine and Molecular Imaging," and "Clinical Nuclear Medicine."

4. **Online Medical and Scientific Databases**: Databases such as PubMed, Scopus, or Web of Science allow you to search for articles related to radiopharmaceutical extravasation and its impact on imaging and therapy in nuclear medicine. Using search terms like "radiopharmaceutical extravasation," "nuclear medicine," and "imaging artifacts" can help you find relevant studies and reviews.

For the most accurate and up-to-date information, consulting these types of sources will be beneficial. Additionally, guidelines and protocols from specific healthcare institutions may also provide insights into how extravasation is managed in clinical practice.

**3^rd^ ChatGPT response**

Radiopharmaceutical extravasation, which is the leakage of a radiopharmaceutical agent into the surrounding tissues at the injection site, can indeed impact the qualitative and quantitative results of a nuclear medicine procedure. This impact can potentially lead to inappropriate or less than optimal care for the patient. The specific effects and the degree to which they can alter patient care depend on several factors, including the type of procedure being conducted, the radiopharmaceutical involved, and the extent of the extravasation. Here are some key considerations:

1. **Quantitative Accuracy**: Extravasation can affect the accuracy of quantitative measurements in nuclear medicine studies. For instance, in procedures like positron emission tomography (PET) or single-photon emission computed tomography (SPECT), extravasation may lead to underestimation of the target organ's uptake of the radiotracer. This underestimation can result in incorrect quantification of physiological or pathological processes, potentially affecting diagnosis and treatment planning.
2. **Image Quality and Interpretation**: Localized accumulation of the radiopharmaceutical in the extravasated area can cause artifacts or abnormal signals on the images. These artifacts may obscure the actual target area or be mistaken for pathological uptake, leading to misinterpretation of the images. In some cases, this can result in incorrect diagnoses or the need for repeat imaging studies, exposing the patient to additional radiation or delays in care.
3. **Treatment Planning and Dosimetry**: For therapeutic nuclear medicine procedures, such as radioiodine therapy for thyroid cancer or peptide receptor radionuclide therapy (PRRT), accurate dosimetry is crucial for effective treatment. Extravasation can lead to inaccurate dosimetry calculations, potentially resulting in suboptimal therapeutic doses to the target tissues or unnecessary radiation exposure to non-target tissues. This can affect treatment efficacy and increase the risk of side effects.
4. **Patient Safety and Comfort**: Apart from affecting the diagnostic or therapeutic outcomes, extravasation can also cause local tissue reactions, pain, or discomfort for the patient. Although most reactions are mild and self-limiting, severe extravasations with certain radiopharmaceuticals may require medical intervention.

To mitigate these risks, nuclear medicine departments implement protocols for preventing, detecting, and managing extravasation. These include careful injection techniques, monitoring the injection site for signs of extravasation, and, if extravasation is detected, taking measures to minimize its extent and potential impact on the study results. In cases where extravasation may significantly affect the procedure's outcome, the study might be rescheduled or additional imaging might be performed to ensure accurate interpretation and patient care.

In summary, radiopharmaceutical extravasation can alter the qualitative and quantitative results of nuclear medicine procedures in ways that might compromise patient care. Awareness, prevention, and management strategies are essential to minimize this risk and ensure optimal patient outcomes.

References

No references were provided- When ChatGPT was asked to provide references, ChatGPT responded as the following:

My response is based on general principles and knowledge within the field of nuclear medicine, as my last training data update was in April 2023, and I don't have the ability to access or cite specific, up-to-date references directly. However, I can guide you on where to find authoritative sources for further reading:

1. **Textbooks on Nuclear Medicine**: Look for comprehensive textbooks that cover the principles of nuclear medicine, radiopharmacy, and the technical aspects of nuclear imaging. These texts often discuss the handling of radiopharmaceuticals, including the prevention and management of extravasation.

2. **Professional Guidelines and Position Statements**: Organizations such as the Society of Nuclear Medicine and Molecular Imaging (SNMMI) in the United States, the European Association of Nuclear Medicine (EANM), and others publish guidelines and position statements on best practices in nuclear medicine. These documents may include sections on radiopharmaceutical injection techniques, extravasation prevention, and management protocols.

3. **Scientific Journals**: Peer-reviewed journals focused on nuclear medicine, radiology, and medical imaging frequently publish research articles, reviews, and case reports on the implications of radiopharmaceutical extravasation. Examples include "The Journal of Nuclear Medicine," "European Journal of Nuclear Medicine and Molecular Imaging," and "Clinical Nuclear Medicine."

4. **Online Medical and Scientific Databases**: Databases such as PubMed, Scopus, or Web of Science allow you to search for articles related to radiopharmaceutical extravasation and its impact on imaging and therapy in nuclear medicine. Using search terms like "radiopharmaceutical extravasation," "nuclear medicine," and "imaging artifacts" can help you find relevant studies and reviews.

For the most accurate and up-to-date information, consulting these types of sources will be beneficial. Additionally, guidelines and protocols from specific healthcare institutions may also provide insights into how extravasation is managed in clinical practice.
