# Supplementary material for: Can ChatGPT help patients understand radiopharmaceutical extravasations?
Source: Front Nucl Med. 2024 Nov 6;4:1469487. doi: 10.3389/fnume.2024.1469487 (PMC11576157; doi:10.3389/fnume.2024.1469487)

**Can ChatGPT Help Patients Understand Radiopharmaceutical Extravasations?**

Madeleine Alvarez^1^

^1^Individual Contributor

*** Correspondence:**Maddie.Alvarez6@gmail.com

Supplemental File 4.


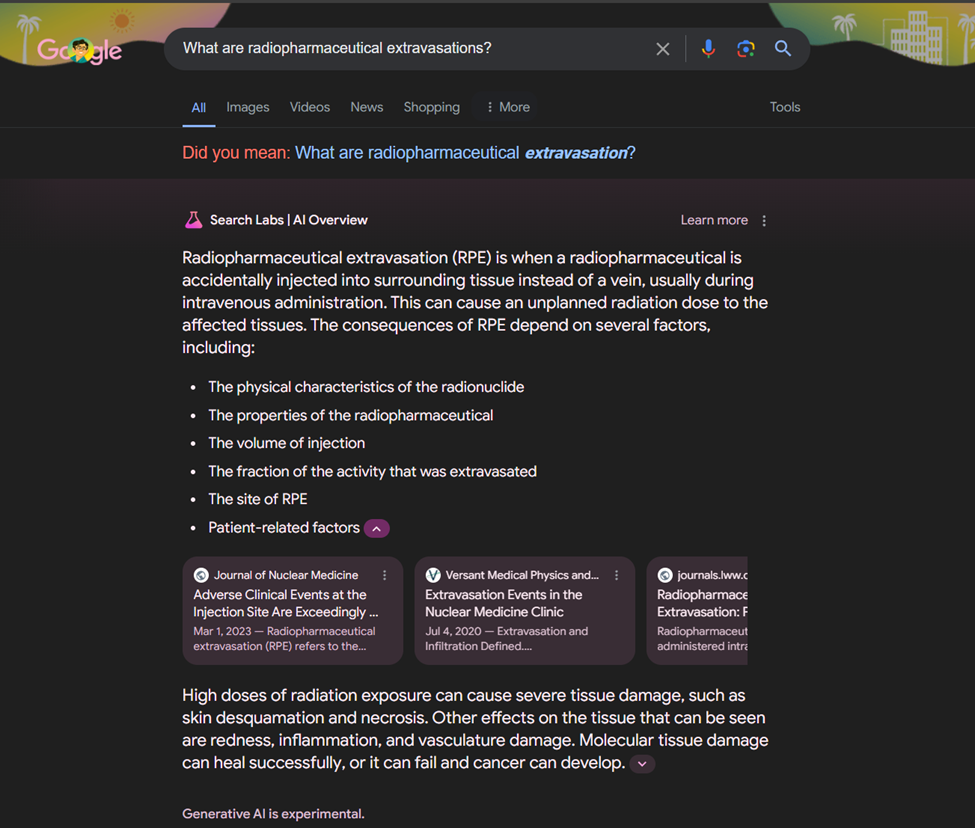

Supplement: Supplementary file 4 [file Table4.docx]
